# Mapping and Validation of *BrGOLDEN*: A Dominant Gene Regulating Carotenoid Accumulation in *Brassica rapa*

**DOI:** 10.3390/ijms232012442

**Published:** 2022-10-18

**Authors:** Lei Zhang, Shifan Zhang, Yun Dai, Shaoxing Wang, Chenggang Wang, Fei Li, Hui Zhang, Guohu Chen, Lingyun Yuan, Jinfeng Hou, Xiaoyan Tang, Shidong Zhu, Rifei Sun, Guoliang Li, Shujiang Zhang

**Affiliations:** 1 Institute of Vegetables and Flowers, Chinese Academy of Agricultural Sciences, Beijing 100081, China; 2 Vegetable Genetics and Breeding Laboratory, College of Horticulture, Anhui Agricultural University, Hefei 230036, China; 3 Beijing Academy of Agricultural and Forestry Sciences, Key Laboratory of Biology and Genetic Improvement of Horticultural Crops (North China), Beijing Key Laboratory of Vegetable Germplasm Improvement, Beijing 100097, China

**Keywords:** carotenoid, *BrGOLDEN*, *Brassica rapa*, spatiotemporal expression, Y2H

## Abstract

In plants, the accumulation of carotenoids can maintain the balance of the photosystem and improve crop nutritional quality. Therefore, the molecular mechanisms underlying carotenoid synthesis and accumulation should be further explored. In this study, carotenoid accumulation differed significantly among parental *Brassica rapa*. Genetic analysis was carried out using the golden inner leaf ‘1900264′ line and the light−yellow inner leaf ‘1900262′ line, showing that the golden inner leaf phenotype was controlled by a single dominant gene. Using bulked−segregant analysis sequencing, *BraA09g007080.3C* encoding the ORANGE protein was selected as a candidate gene. Sequence alignment revealed that a 4.67 kb long terminal repeat insertion in the third exon of the *BrGOLDEN* resulted in three alternatively spliced transcripts. The spatiotemporal expression results indicated that *BrGOLDEN* might regulate the expression levels of carotenoid−synthesis−related genes. After transforming *BrGOLDEN* into *Arabidopsis thaliana*, the seed−derived callus showed that *BrGOLDEN_Ins_* and *BrGOLDEN_Del_* lines presented a yellow color and the *BrGOLDEN_Ldel_* line presented a transparent phenotype. In addition, using the yeast two−hybrid assay, BrGOLDEN_Ins_, BrGOLDEN_Ldel_, and Brgolden_wt_ exhibited strong interactions with BrPSY1, but BrGOLDEN_Del_ did not interact with BrPSY1 in the split−ubiquitin membrane system. In the secondary and 3D structure analysis, BrGOLDEN_Del_ was shown to have lost the PNFPSFIPFLPPL sequences at the 125 amino acid position, which resulted in the α−helices of BrGOLDEN_Del_ being disrupted, restricting the formation of the 3D structure and affecting the functions of the protein. These findings may provide new insights into the regulation of carotenoid synthesis in *B*. *rapa*.

## 1. Introduction

Carotenoids play a fundamental role in human nutrition by maintaining human health and mitigating a range of diseases [1]. They are derived from isoprenoids, the largest class of natural pigments in plants. To date, more than 750 natural carotenoid molecules have been identified from animals, plants, and microorganisms [2]. In plants, carotenoids protect the photosystem from damage by dissipating excess light energy released by the photosynthetic mechanism [3]. Carotenoids can also serve as precursors for the phytohormone strigolactone and abscisic acid biosynthesis [4,5,6,7]. In edible fleshy fruit, such as cantaloupe, melon, and citrus, carotenoid volatile derivatives affect the aroma and flavor [8]. Moreover, the activity of reactive oxygen species and free radicals can be inhibited by carotenoids to maintain cell structure and normal life activities [9]. Therefore, improving the carotenoid content in plants has attracted increasing attention, especially the content of carotenoids in fruit, vegetables, and grains, which are the main components in the human diet. In recent years, considerable details of the plant carotenoid biosynthetic pathway have been elucidated [10,11,12].

Carotenoids can be produced in all differentiated plastids and mainly accumulate in the chloroplasts of green tissues and chromoplasts of petals and fruit [13,14]. In plants, many core catalytic reaction genes and enzymes for carotenoid synthesis have been identified (Figure 7e) [2]. Carotenoids are formed from the condensation of the 5−carbon precursors isopentenyl diphosphate and dimethylallyl diphosphate, which are produced via the plastidial 2−C−methyl−D−erythritol 4−phosphate (MEP) pathway in plastids [15]. The first committed step in the carotenoid pathway occurs when phytoene synthase (*PSY*) condenses two molecules of geranylgeranyl diphosphate (*GGPP*) to form 15−*cis*−phytoene [16]. *PSY* is considered the rate−limiting enzyme that determines carotenoid synthesis. 15−*Cis*−phytoene is transformed into trans−lycopene under the catalysis of phytoene desaturase (*PDS*) and ζ−carotene desaturase (*ZDS*). The next step can follow two paths: α−carotene is formed via lycopene ε−cyclase (*ε*−*LCY*) and β−carotene is formed via *β*−*LCY* [17,18,19,20]. Subsequently, α− and β−carotene are catalyzed by heme−containing cytochrome P450−type hydroxylases (*CYP*) and non−heme β−ring hydroxylases (*BCH*), respectively, to hydroxylate the distal ring structure to generate yellow lutein and zeaxanthin. Zeaxanthin can be epoxidized by zeaxanthin epoxidase (*ZEP*) to produce violaxanthin, which can be reversed by violaxanthin de−epoxidase (*VDE*). This interconversion forms the xanthophyll cycle. Finally, neoxanthin is produced by neoxanthin synthase (*NXS*), completing the main biosynthetic pathway of carotenoids [21,22,23]. These carotenoids are indispensable for their role in catalyzing the production of strigolactone, caprolactone, β−citraurin, volatiles, and scents. Thus far, in *Arabidopsis thaliana* [24] and many horticultural plants, such as *Daucus carota* [25], *Solanum lycopersicum* [26,27], *Solanum tuberosum* [28], *Capsicum annuum* [29], and *Brassica rapa* [30], there has been great progress in the study of carotenoid metabolism.

Chinese cabbage (*B. rapa*) is a leafy vegetable belonging to the *Brassica* genus in the cruciferous family [31]. Its cultivated area and consumption rank first among all vegetables in China. The inner leaves of Chinese cabbage are rich in carotenoids, which are responsible for the diverse colors of Chinese cabbage leaves. The abnormal accumulation of carotenoids in Chinese cabbage with orange heads is mostly controlled by a series of recessive genes [32]. Matsumoto et al. (1998) developed rapid fragment length polymorphism (RFLP) markers for *orange*−*yellow* gene (*Oy*) pigmentation in Chinese cabbage and mapped *Oy* on linkage group 1. Genetic analysis has indicated that *Oy* is a recessive gene [33]. Using bulked segregant analysis (BSA)−seq and sequence characterized amplified region (SCAR) markers, localization analysis of the orange inner leaf trait of Chinese cabbage was carried out and 110 Chinese cabbage samples were verified using the three developed SCAR markers. The candidate gene was located on the R9 linkage group [34]. Feng et al. (2012) used 600 individual plants from the F2 population to map and develop new markers closely related to the genes for the orange color on the inner leaves of Chinese cabbage. Using linkage analysis, a gene was located at the end of A09 and the distance between the markers *syau19* and *syau15* was found to be 4.6 cM [32]. In addition, the *orange* locus was finely mapped in the F_2_ S_4_ populations and *ORF1*, a candidate gene, encodes a carotenoid isomerase, involved in the isomerization of carotenoids. Sequencing analysis has revealed insertions/deletions in the *ORF1* promoter region between the two parents. Therefore, *CRTISO* (*carotene isomerase*) is the most likely candidate gene for *Bror* [35]. Lee et al. (2014) found a mutation in a gene encoding *CRTISO* in an orange−colored Chinese cabbage and verified several molecular markers that could distinguish *CRTISO* genotypes. This was the first time that *CRTISO* has been identified as a candidate gene in the OC inner leaves of Chinese cabbage [36]. In subsequent studies, genetic analysis and fine mapping were conducted in the orange inner leaves of Chinese cabbage from different parents, indicating that *BrCRTISO* is a single recessive candidate gene. In the parental material, *BrCRTISO* has many Indels and single nucleotide polymorphisms (SNPs) in the promoter and coding regions, resulting in orange inner leaves in Chinese cabbage [37,38,39].

To date, many recessive genes regulating carotenoid accumulation in *B*. *rapa* have been discovered, but no dominant gene controlling this trait has been found. In this study, *BrGOLDEN*, a dominant gene, was mapped and cloned to regulate carotenoid accumulation in *B*. *rapa*. Tri−crossed hybrid lines were constructed through hybridization between the *B*. *rapa* line with golden inner leaves and the non−golden highly inbred line. Carotenoid pigments were identified by high−performance liquid chromatography (HPLC) in the leaves of different *B*. *rapa* lines. In addition, the promoter and coding sequences of the candidate genes were cloned and transformed into *A. thaliana* to verify their functions. The interaction proteins were screened by Yeast Two−Hybrid (Y2H), which revealed that *PSY* played an important role in regulating carotenoids and different transcripts of *BrGOLDEN* were selected. Meanwhile, quantitative real−time (qRT)−PCR was used to analyze the expression of the candidate genes and genes in the carotenoid regulation pathways, indicating how *BrGOLDEN* influences other genes. This study lays a foundation for the elucidation of the molecular mechanisms of carotenoid biosynthesis in the golden inner leaves of the *B*. *rapa* line, which may help determine the carotenoid regulation pathways.

## 2. Results

### 2.1. Phenotypic Evaluation and Genetic Analysis

**Figure 1:**
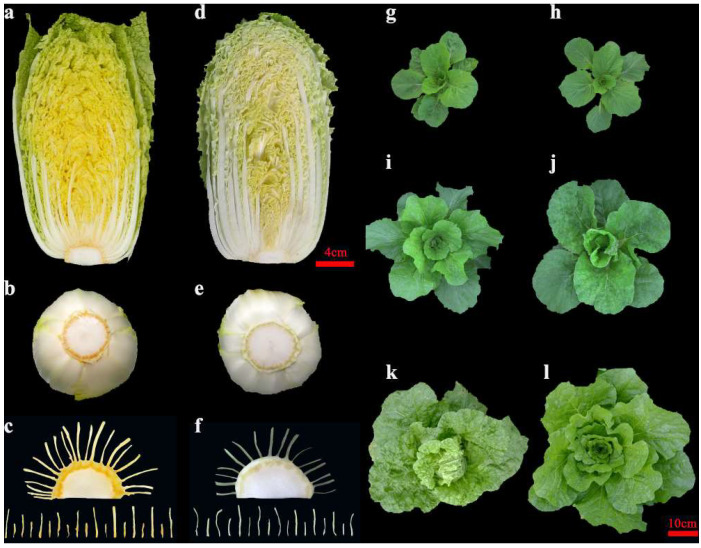
Morphological observation of two *Brassica rapa* lines. Sectional view of the ‘1900264′ line (**a**) and ‘1900262′ line (**d**) at the mature stage (14 weeks). Phenotypes of short stems in the ‘1900264′ line (**b**,**c**) and the ‘1900262′ line (**e**,**f**) at the mature stage (14 weeks). Phenotype of the ‘1900264′ line grown for 4 (**g**), 6 (**i**), and 10 (**k**) weeks. Phenotype of the ‘1900262′ line grown for 4 (**h**), 6 (**j**), and 10 (**l**) weeks.

Line ‘1900264′, a commercial *B*. *rapa* variety, is a male−sterile hybrid line (P1, called F_1_, parental information is unknown) with golden inner leaves (Figure 1a–c). Line ‘1900262′ (P2) is a highly inbred *B*. *rapa* line that has non−golden inner leaves and no significant carotenoid accumulation in the short stem tissue (Figure 1d–f). The morphology and growth of the two Chinese cabbage lines are similar in the seedling and rosette stages (Figure 1g–j). However, compared with line ‘1900264′, line ‘1900262′ is an early heading material, with a higher degree of heading and relatively curly leaves (Figure 1k,l). The tri−crossed hybrid lines were obtained through ‘1900264′ × ‘1900262′. Then, the inner leaf phenotypes of the tri−crossed hybrid line were investigated and counted and the golden and non−golden phenotypes conformed to a 1:1 segregation ratio at the Chi−squared test **(**χ^2^ test) level (Table 1). Therefore, the golden inner leaf phenotype was controlled by a pair of dominant nuclear genes, which was named *BrGOLDEN*. The phenotypic investigation also showed that the ‘golden circle’ appeared in Chinese cabbage with golden inner leaves; we speculate that the ‘golden circle’ in short stem tissue may be linked to the golden inner leaf phenotype (Figure 1a–f).

### 2.2. Comparison of Microstructure and the Carotenoid Component

**Figure 2:**
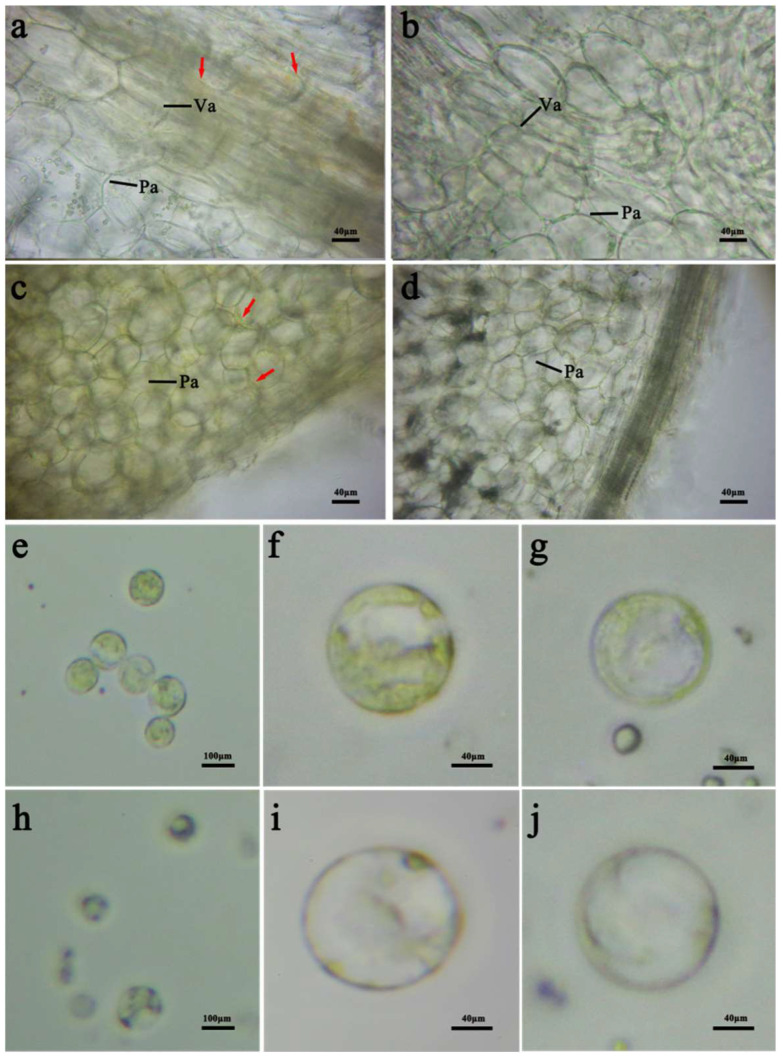
Microstructure of the inner leaves and short stems in the ‘1900262′ and ‘1900264′ lines. Microstructure of the short stem cells in the ‘1900264′ (**a**) and ‘1900262′ lines (**b**). Microstructure of the inner leaf cells in the ‘1900264′ (**c**) and ‘1900262′ lines (**d**). Inner leaf protoplasts of the ‘1900264′ (**e**) and ‘1900262′ lines (**h**) under 10× light microscope. Inner leaf protoplasts of the ‘1900264′ (**f**) and ‘1900262′ lines (**i**) under 40× light microscope. Short stem protoplasts of the ‘1900264′ (**g**) and ‘1900262′ lines (**j**) under 40× light microscope. The point of the red arrow indicates the chromoplasts. Va: vascular bundle; Pa: parenchyma cell.

The microstructures of the short stems and the inner leaves at the maturity stage were analyzed by observing microsection images of the ‘1900264′ and ‘1900262′ lines. In line ‘1900264′, more chromoplasts were aggregated in the xylem cells of the short stem, but fewer chromoplasts were produced in the parenchyma cells (Figure 2a) and, in the inner leaves, the golden chromoplasts were distributed around all cells (Figure 2c). However, this distribution of chromoplasts was not observed in the cells of the short stem and the inner leaves in the ‘1900262′ line, whose inner leaves were the non−golden phenotype (Figure 2b,d).

The protoplasts were separated from the short stem and inner leaf tissues of the parent lines and observed under a light microscope. Many chromoplasts were distributed in the short stem and inner leaf cells of the ‘1900264′ line (Figure 2e–g). Chromoplasts were not observed in the short stems of the non−golden inner leaves of the ‘1900262′ line, but a few chromoplasts were found in the protoplasts of the inner leaves (Figure 2h–j). This may be because the ‘1900262′ line has light yellow inner leaves and a low carotenoid content; thus, the presence of a few chromoplasts was observed. Thus, the chromoplasts contained in these cells may be related to the color of the short stems and the inner leaves in the golden *B*. *rapa* line.

**Figure 3:**
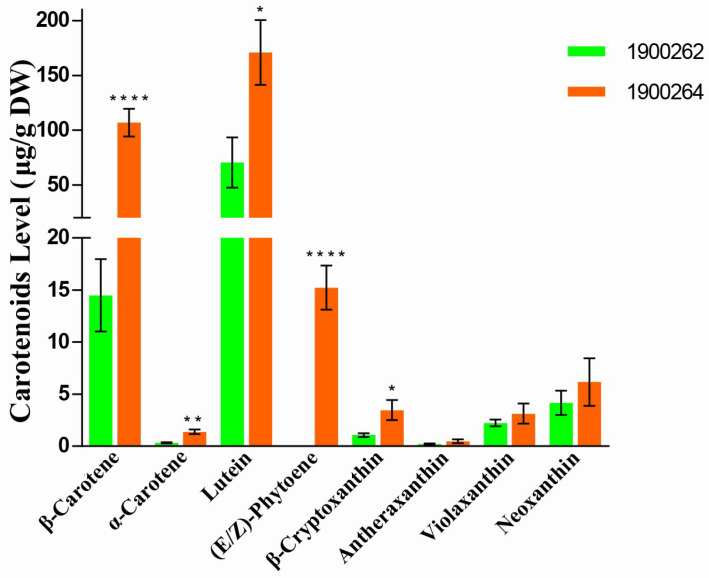
Comparison of carotenoid content in the inner leaves of the ‘1900262′ and ‘1900264′ lines. Data are the average ± SE of three biological replicates. Asterisk (*) indicates that there is a statistical difference, * indicates *p* ≤ 0.05; ** indicates *p* ≤ 0.01; **** indicates *p* ≤ 0.0001.

To analyze the carotenoid compositions of the inner leaves, the 5 cm long inner leaves of the two parent lines (‘1900264′ and ‘1900262′ lines) at the mature stage were used to detect carotenoid components and content. Using the liquid chromatography−tandem mass spectrometry (LC−MS/MS) platform, 20 and 46 carotenoid components were subjected to absolute and relative quantification, respectively (Table S1). Among them, eight components were primarily detected and the contents of the other components were relatively low. Eight carotenoid components were detected. The total carotenoid content of the ‘1900264′ line was 3.3−times that of the ‘1900262′ line (Figure 3). Except for neoxanthin, violaxanthin, and antheraxanthin, the contents of the other five carotene components differed between the ‘1900264′ and ‘1900262′ lines. The ‘1900264′ line was rich in β−carotene and lutein, which accounted for 35.2% and 56.2% of the total carotenoid content, respectively. Compared with the ‘1900262′ line, the content of β−carotene increased by 13.6−fold. Interestingly, colorless (E/Z)−phytoene, as the upstream product of carotenoid biosynthesis [40], was detected only in the ‘1900264′ line.

### 2.3. Primary Mapping for the Golden Inner Leaf Phenotype by BSA−seq

**Figure 4:**
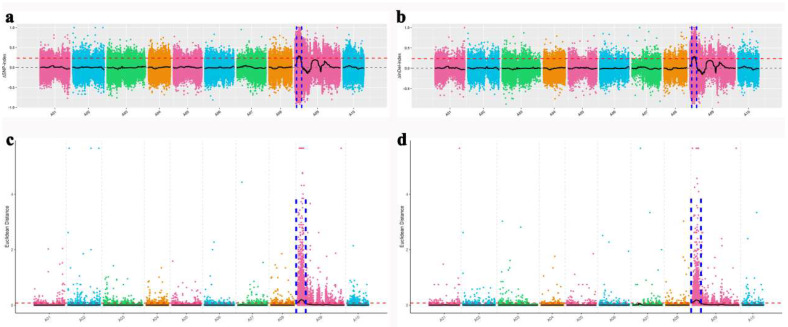
Genetic mapping of the golden inner leaf phenotype in *B*. *rapa*. Identification of the *BrGOLDEN* candidate region through the △SNP index (**a**) and △InDel index; (**b**) association analysis method. *BrGOLDEN* candidate regions identified by Euclidean distance (ED) and SNP (**c**) or ED and InDel (**d**) association analysis. The higher the index value or ED value, the better the association effect.

The BSA method can rapidly identify markers linked to any specific gene or genomic region [41]. Forty golden and non−golden individuals were selected from the tri−crossed hybrid lines to construct the golden pool (GP) and non−golden pool (NGP), respectively. After resequencing, quality control, assembly, and alignment with the *B*. *rapa* reference genome V3.0 (http://brassicadb.org, accessed on 10 February 2021), the GP contained 179.87 Mb reads (98.39% coverage) and the NGP contained 180.56 Mb reads (98.41% coverage) (Table S2). Using association analysis of the Euclidean distance (ED) algorithm [42] and the △index algorithm [43], the intersection association area corresponding to all SNPs and Indels was obtained. Finally, *BrGOLDEN* was primarily located in the 2.2 Mb region of A09 (Figure 4).

### 2.4. Verifying the BrGOLDEN Candidate Gene

Based on the BSA−seq mapping results, the candidate region of 2.2 Mb was identified in combination with the *B. rapa* and *A. thaliana* databases. A total of 532 genes was annotated within the candidate region (Table S4). The *BraA09g007080.3C* at the anterior end of A09 was homologous to *AT5G61670* (*AtOR*) in *A. thaliana*. In cauliflower, *BoOR* is a dominant gene regulating carotene accumulation [44]. Similar to *AtOR*, *BraA09g007080.3 C* contained a DnaJ−like zinc finger domain. In a previous study, *AtOR* interacted directly with *PSY* (phytoene synthase), which acts as a positive posttranscriptional regulator to control carotenoid biosynthesis [45,46]. Thus, *BraA09g007080*.*3C* is likely the candidate gene *BrGOLDEN* associated with inner leaf color.

**Figure 5:**
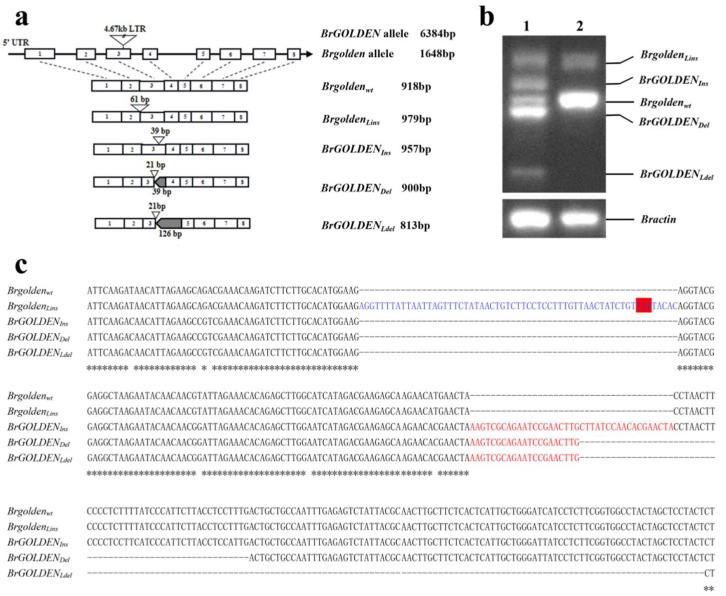
*BrGOLDEN* structure analysis. (**a**) The *BrGOLDEN* with the 4.67 kb LTR insertion site and different transcripts. Open boxes with numbers represent exons and solid lines represent introns. Triangles indicate insertion sites and grey boxes indicate deletion sites. *Brgolden_Lins_*, large insertion; *BrGOLDEN_Ins_*, insertion; *BrGOLDEN_Del_*, deletion; *BrGOLDEN_Ldel_*, large deletion. (**b**) RT−PCR amplification of *BrGOLDEN* in the ‘1900264′ (lane 1) and ‘1900262′ lines (lane 2) cDNAs. *Bractin* served as an internal control. (**c**) Nucleotide sequence alignment of five transcript fragments. The blue words represent the transcripts resulting from the complete insertion of the second intron in the ‘1900262′ line and the red box indicates the translation end site. The red words and dotted lines indicate the splicing patterns of the three transcripts unique to the ‘1900264′ line.

To analyze the *BraA09g007080*.*3C* sequences of possible candidate genes in the ‘1900264′ and ‘1900262′ lines, the gDNA and CDS were amplified and sequenced using specific primers (Table S4). In line ‘1900262′, the gDNA of the *Brgolden* was 1648 bp, contained eight exons and seven introns, and two transcripts were obtained from it. *Brgolden_wt_* was 918 bp and encoded a protein with 305 amino acids. *Brgolden_Lins_* (Lins, large insert transcript), with the second intron (61 bp) fully inserted, results in a frameshift mutation that prematurely terminates translation and, presumably, this transcript may be non−functional (Figure 5a). The reverse transcription (RT)−PCR results showed that *Brgolden_wt_* was more abundant than *Brgolden_Lins_* in the ‘1900262′ line (Figure 5b). In line ‘1900264′, sequence analysis of *BrGOLDEN* showed that a large fragment of 4.67 kb was inserted at 558 bp and the insertion event led to the production of three alternative splicing transcripts. During the transcription process, only 21 bp remained at the 5′ end of the large insertion fragment. *BrGOLDEN_Ins_* (Ins, insert transcript) contained an additional 18 bp insertion in the third exon. *BrGOLDEN_Del_* (Del, deletion transcript) contained a 39 bp deletion in the third exon and *BrGOLDEN_Ldel_* (Ldel, large deletion transcript) contained a 126 bp large deletion in the third and fourth exons (Figure 5a,c). The relative expression level of *BrGOLDEN_Del_* was the most abundant in the ‘1900264′ line (Figure 5b). Amino acid sequence alignment showed that these transcripts were highly homologous and all contained four conserved C××C×××G motifs (Figure S1). Thus, we propose that *BraA09g007080*.*3C* is the candidate gene for golden and non−golden inner leaf color in Chinese cabbage.

**Figure 6:**
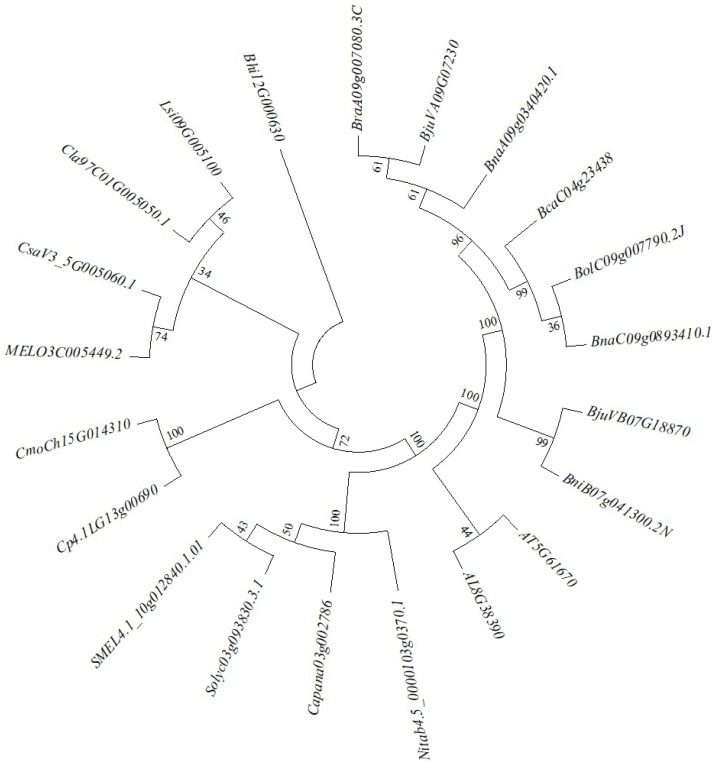
Phylogenetic analysis of *ORANGE* in Cruciferae and other species. The phylogenetic tree was constructed in MEGA 7.0 software (Mega Limited, Auckland, New Zealand) using the neighbor−joining method. The bootstrap replication values were determined from 1000 replicates.

The *OR* homologous gene *BrGOLDEN* in *B*. *rapa* was selected as a candidate gene for regulating carotenoid accumulation. OR is unique to plants and there were obvious differences among various plant families [44]. To analyze the evolutionary relationships of *BrGOLDEN* in horticultural crops, the sequences were aligned in different species. A highly conserved Cys−rich DnaJ domain was present in Cruciferae, Cucurbitaceae, and Solanaceae, among others. Phylogenetic analysis showed that *B*. *rapa* (*BraA09g007080.3C*) was most closely related to *Brassica juncea*, *Brassica napus*, *Brassica oleracea*, and *Brassica carinata* (Figure 6).

### 2.5. Relative Expression Level Analysis of BrGOLDEN and Carotenoid Synthesis Pathway Genes

**Figure 7:**
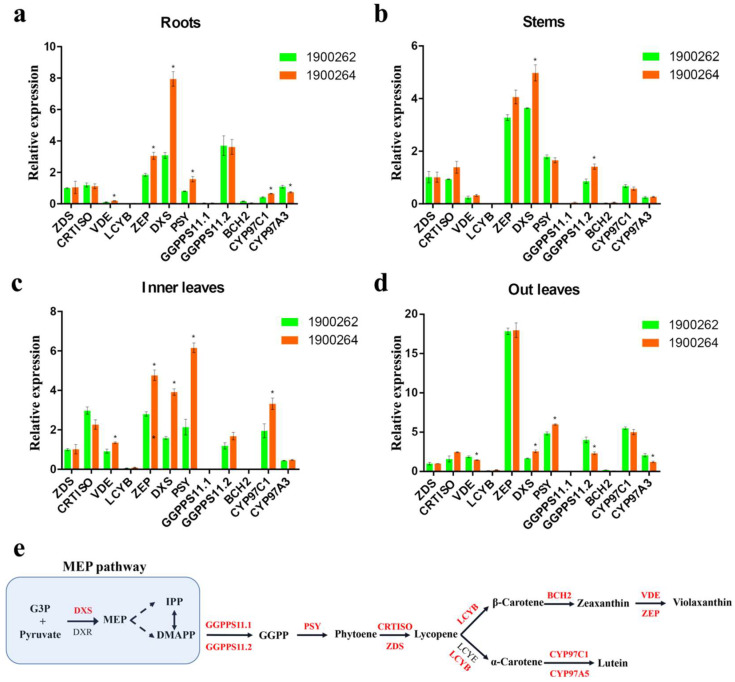
Expression of carotenoid biosynthetic genes in lines ‘1900262′ and ‘1900264′. Relative expression levels of carotenoid−synthesis−related genes in the roots (**a**), short stems (**b**), inner leaves (**c**), and outer leaves (**d**) of lines ‘1900262′ and ‘1900264′. *ZDS*, *BraA05g040310.3C*; *CRTISO*, *BraA09g063710.3C*; *VDE*, *BraA06g005700.3C*; *LCYB*, *BraA03g017230.3C*; *ZEP*, *BraA07g016880.3C* and *BraA07g016890.3C*; *DXS*, *BraA01g021140.3C*; PSY, *BraA02g006890.3C*; *GGPPS11.1*, *BraA08g021280.3C*; *GGPPS11.2*, *BraA01g002000.3C*; *BCH2*, *BraA10g010930.3C*; *CYP97C1*, *BraA07g021850.3.5C*; *CYP97A3*, *BraA08g009810.3C*. An asterisk (*) indicates that there is a statistical difference, *p* ≤ 0.05, *n* = 3. *Bractin* served as a control. (**e**) Outline of the carotenoid biosynthetic pathway. Red words indicate genes validated in the experiment.

To investigate whether *BrGOLDEN* promoted carotenoid accumulation by affecting the expression of carotenoid−synthesis genes, the relative expression levels were analyzed, including the *BrGOLDEN/Brgolden* and 12 carotenoid synthesis pathway genes (*ZDS*, *CRTISO*, *VDE*, *LCYB*, *ZEP*, *DXS*, *PSY*, *GGPPS11*.*1*, *GGPPS11*.*2*, *BCH2*, *CYP97C1*, and *CYP97A3*) (Figure 7e). The proteins encoded by these genes were extensively involved in carotenoid synthesis and catalytically produced carotenoid components were also significantly detected. RNA was extracted and reverse transcribed into cDNA from four tissues (the outer leaves (OL), inner leaves (IL), short stems (S), and roots (R)) and in three periods (seedling, rosette, and heading stages) in the ‘1900262′ and ‘1900264′ lines. Compared with *Brgolden*, the relative expression levels of *BrGOLDEN* were not significantly higher, in the four tissues from the three different periods (Figure S2a). Compared with IL, S, and R, the relative expression levels of *BrGOLDEN* and *Brgolden* in OL were highest (Figure S2a,b). Twelve key genes of the carotenoid synthesis pathway were analyzed at maturity. Compared with line ‘1900262′, the relative expression level of *DXS* was significantly higher in the roots of line ‘1900264′ (Figure 7a). However, these genes were not significantly different in the short stems of either line (Figure 7b). In particular, the relative expression levels of four genes (*ZEP*, *DXS*, *CYP97C1*, and *PSY*) were higher in the inner leaves of the ‘1900264′ line than in the ‘1900262′ line (Figure 7c). In addition, the relative expression levels of *ZEP* were similar between lines ‘1900262′ and ‘1900264′ but were significantly higher than the other 11 genes in the outer leaves (Figure 7d). Previous studies found similar transcript levels of carotenoid biosynthesis genes in cauliflower and *A*. *thaliana* [47,48]. Thus, *BrGOLDEN* may promote carotenoid accumulation by regulating the expression of genes in the carotenoid synthesis pathway in *B*. *rapa*.

### 2.6. Brgolden Exhibits Distinct Expression Patterns

**Figure 8:**
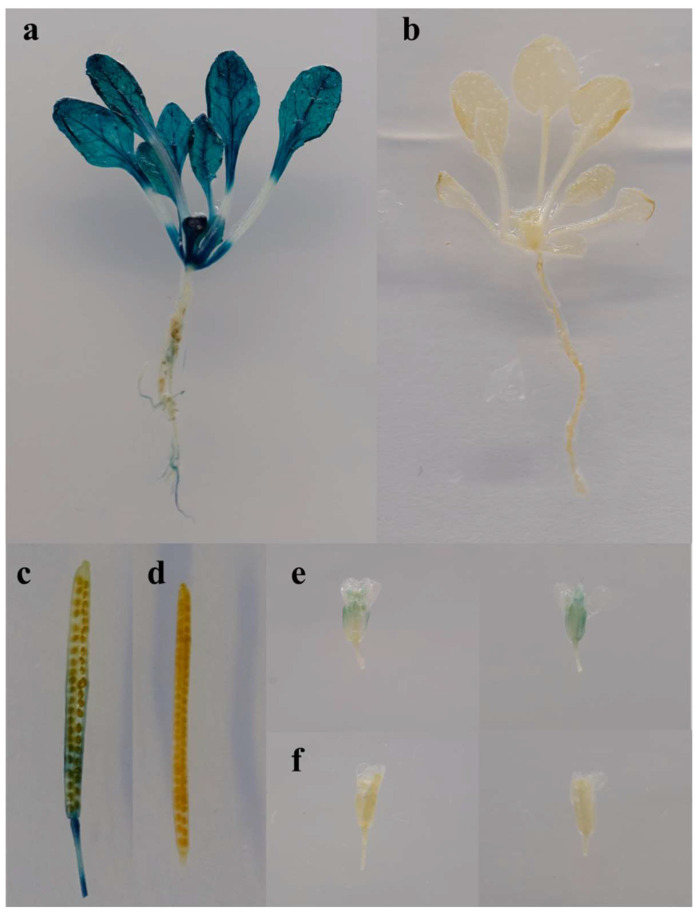
β−Galactosidase (GUS) staining of different tissues of Pro*_Brgolden_*:GUS transgenic *Arabidopsis thaliana*. Three−week−old seedlings of Pro*_Brgolden_*: GUS (**a**) and wild−type lines (**b**). Mature silique of Pro*_Brgolden_*: GUS (**c**) and wild−type lines (**d**). Flowers of Pro*_Brgolden_*: GUS (**e**) and wild−type lines (**f**).

The *cis*−acting element of the *Brgolden* promoter was predicted and transgenic plants with a β−galactosidase (GUS) reporter driven by the *Brgolden* promoter (Pro*_Brgolden_*:GUS) were generated in *A*. *thaliana*. Basic elements, such as central promoter elements (TATA−box) and enhancer elements (CAAT−box) necessary for eukaryotes, were included in the *Brgolden* promoter. In addition, there were several specific regulatory elements, including five light−responsive−related elements (GATA−motif, Box 4, TCT−motif, I−box, and G−box), two types of hormone−responsive *cis*−elements (TCA−element and ABRE), elements required for anaerobic induction (ARE), and a specific element of the roots (motif I) and seeds (RY−element) (Table 2). The GUS staining results of the three−week−old transgenic *A*. *thaliana* plants showed that GUS was expressed in the leaves, leaf veins, and petioles, but less so in the roots (Figure 8a,b). At the mature stage, there was higher GUS activity in the calyx, stigma, seed pods, and the junctions between the seed pods and seed stalks, whereas there was less GUS expression in the petals (Figure 8c–f). In summary, the *Brgolden* promoter may be a constitutive promoter driving *GUS* expression in different tissues.

### 2.7. Ectopic Overexpression of BrGOLDEN Increases Carotenoid Accumulation in A. thaliana Calluses

**Figure 9:**
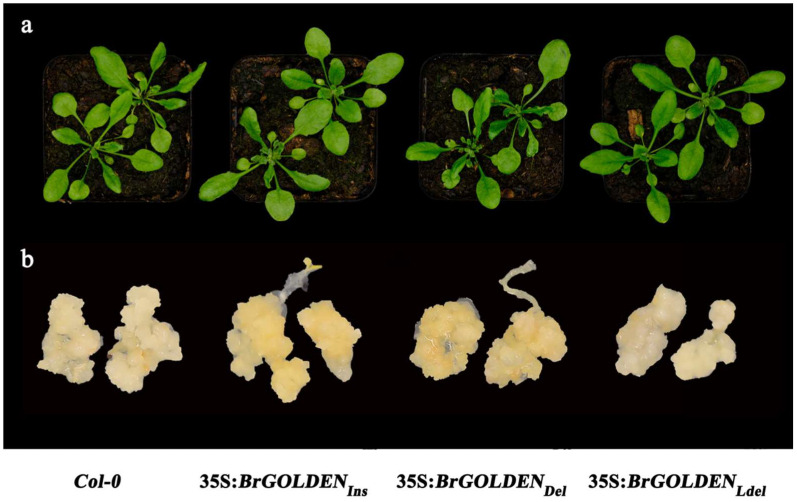
Phenotypic observations of plants and seed−derived calluses of *A. thaliana* lines overexpressing *BrGOLDEN* lines. (**a**) Rosette stage phenotypes of 35 S:*BrGOLDEN* transgenic and wild−type *A. thaliana* seedlings at four weeks. (**b**) Representative images from seed−derived calluses of *BrGOLDEN* overexpressing lines and the wild type.

The function of *BrGOLDEN* was analyzed by constructing 35S:*BrGOLDEN* overexpressing (OE) vectors and transforming them into the *A*. *thaliana* wild type (*Col−*0). From the obtained transgenic lines, one OE representative line of each alternative splicing transcript (35S:*BrGOLDEN_Ins_*, 35S:*BrGOLDEN_Del_*, and 35S:*BrGOLDEN_Ldel_*) was selected for the observation of phenotypic changes. All transgenic *A*. *thaliana* mutants were similar to the wild type in plant morphology and leaf phenotype (Figure 9a), which was consistent with the results of the wild type and *BoOR_MUT_ B*. *oleracea* [47]. This also indicates that carotenoid composition and content are in a dynamic balance in the leaf tissue for optimal photosynthesis [49]. Compared with the leaves, non−green tissues, such as calluses, tend to respond phenotypically to an increased carotenoid content [50]. In recent years, calluses have been used to rapidly characterize the function of genes involved in carotenoid biosynthesis and accumulation [51]. Therefore, calluses were induced from the seeds of these overexpressing *A*. *thaliana* lines and the wild type (Figure 9b). Under the same conditions, the calluses induced by 35S:*BrGOLDEN_Ldel_* and the wild type were nearly transparent or white, while the calluses of *BrGOLDEN_Ins_* and *BrGOLDEN_Del_* were yellow, inferring that there was an accumulation of carotenoids. Thus, the function of *BrGOLDEN* was determined by different spliced transcripts.

### 2.8. Identification of the Target Proteins Involved in BrGOLDEN Interactions

**Figure 10:**
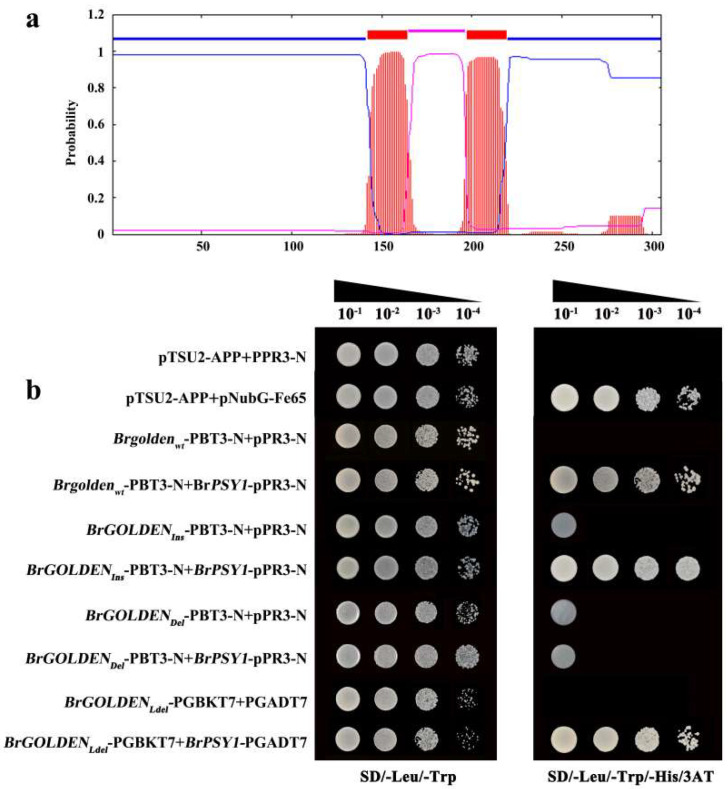
Interactions of BrPSY1 with BrGOLDEN. (**a**) Prediction of transmembrane helices in Brgolden_wt_. The red line represents the transmembrane. The blue line represents the inside. The pink line represents the outside. Positions 142–164 and 197–219 amino acids represent transmembrane helices. The abscissa indicates the amino acid site. (**b**) Co−transformation of prey and bait vectors in various combinations validates protein interactions. Co−transformation of pPR3−N and pTSU2−App vectors was used as a negative control and co−transformation of pTSU2−APP and pNubG−Fe65 vectors was used as a positive control. Dilutions (1–10^−4^) of different concentrations of the saturated cultures were spotted onto SD/−Leu/−Trp/−His/−3AT plates.

PSY is the rate−limiting enzyme in the carotenoid synthesis pathway and its activity greatly affects carotenoid accumulation. Previous studies have shown that the N−terminus of OR physically interacts with PSY and positively regulates the PSY level and enzymatic activity, while the C−terminus forms a dimeric structure [46,52]. Treatment with the protein synthesis inhibitor cycloheximide (CHX), OR, was found to stabilize PSY, greatly reduce the protein turnover rate, and significantly increase carotenoid levels [53]. In this study, Brgolden_wt_ was a double−transmembrane protein (Figure 10a). Due to the different splicing modes of the N−terminus of the three alternative splicing transcripts, the integrity of the N−terminal and the transmembrane domain was destroyed (Figure S3). Therefore, the split−ubiquitin membrane Y2H system was used to verify whether Brgolden_wt_, BrGOLDEN_Ins_, and BrGOLDEN_Del_ interacted with BrPSY1. The interaction between BrGOLDEN_Ldel_ and BrPSY1 was verified using Y2H based on the nuclear interaction system (Figure 10b). Interestingly, although BrGOLDEN_Ins_, BrGOLDEN_Ldel_, and Brgolden_wt_ exhibited strong interactions with BrPSY1, BrGOLDEN_Del_ did not interact with BrPSY1 in the split−ubiquitin membrane system. In addition, after BrGOLDEN_Del_−PBT3−N and BrPSY1−pPR3−N co−transformed yeast strains through the membrane interaction system, the negative and positive controls had the expected results. The strain on the DDO plate grew well, indicating that it was successfully transferred into the host cells. However, they did not grow on either the QDO or TDO plates supplemented with 5 mM 3AT, which was verified with replication. There was no interaction between BrGOLDEN_Del_−PBT3−N and BrPSY1−pPR3−N in the split−ubiquitin membrane system. The amino acid deletion of BrGOLDEN_Del_ played an important role in its interaction with BrPSY1.

Compared with Brgolden_wt_, the amino acid sequence of BrGOLDEN_Del_ lost the PNFPSFIPFLPPL sequence at position 125 amino acids and was replaced by the sequence KSQNPNL (Figure S1). The protein secondary structures of BrGOLDEN_Del_ and Brgolden_wt_ were predicted by Phyre2 (Figure S4) and included 11 *β*−strands and seven and nine *α*−helices, respectively. Due to sequence deletions, the important α−helix of the secondary structure of BrGOLDEN_Del_ was disrupted. The missing protein structure is located on the surface of the 3D structure, which plays an important role in the interactions between BrGOLDEN and BrPSY1. Using 3D structure prediction analysis, the Brgolden_wt_ structure was predicted successfully and BrGOLDEN_Del_ was not, which indicated that the loss of the sequences at position 125 amino acids limited its function. In addition, by analyzing the 3D structure of Brgolden_wt_, this protein may be a tryptophan RNA−binding attenuator protein inhibitory protein with three molecular functions (binding, protein binding, and identical protein binding) and three cellular components (intracellular anatomical structure, cytoplasm, and cellular anatomical entity) [54]. This study further verified that the 3D structure of the protein was the most direct factor affecting the functions of the protein. Due to the mutation of several amino acids in BrGOLDEN_Del_, the 3D structure of this protein was greatly changed, affecting the interaction between BrGOLDEN_Del_ and BrPSY1.

## 3. Discussion

The synthesis of carotenoids is very important in plants and the various 40−carbon compounds produced by metabolic pathways provide plants with red, orange, and yellow colors and give them ornamental value. The varied inner leaf colors of Chinese cabbage are attributed to different carotenoid compositions and concentrations. Due to the nutritional and health−related properties of carotenoids, research on carotenoid metabolism in Chinese cabbage inner leaves is an active field. At the same time, carotenoids are widely involved in growth and development processes, such as photosynthesis and plant stress. Studies have shown that carotenoids are synthesized in chromoplasts and are mainly found in non−green tissues [55]. In this study, a dominant gene *BrGOLDEN*, controlling carotenoid accumulation, was identified for the first time in the Chinese cabbage line with golden inner leaves. The results showed that *BraA09g007080*.*3C*, which was homologous to *AtOR*, was considered a candidate gene for *BrGOLDEN*. ORANGE is an important protein that stabilizes the rate−limiting enzyme PSY for carotenoid synthesis, which converts GGPPs to phytone [56,57]. As PSY protein level is crucial for overall pathway activity and carotenoid amounts, a tight control of PSY proteostasis is expected. Studies have showed that OR can also regulate the plastid−localized protein degradation machinery, namely the Clp protease complex, to maintain PSY proteostasis and fine−tune carotenogenesis [46,53].

In a previous study, the homolog *BoOR* of *BrGOLDEN* was reported in *B*. *oleracea* [44]. Comparing the sequences of *BrGOLDEN* with *BoOR*, an insertion event and three alternative splicing transcripts of *BrGOLDEN* were very similar to the *BoOR* mutant (Figures S5 and S6). In *B*. *oleracea*, a large fragment from C06 was inserted into *BoOR* in C09 and an extra 16 bp was generated, resulting in a 4686 bp fragment insertion. Unlike *BoOR*, *BrGOLDEN* retained only the entire insertion fragment sequence (4670 bp). RT−PCR was used to detect regulatory elements, including the *CaMV* 35S promoter, *FMV* 35S promoter, *NOS* promoter, *NOS* terminator, and *CaMV* 35S terminator, according to the testing method for genetically modified new varieties (Ministry of Agriculture and Rural Affairs of the People’s Republic of China) and none of the target amplification products were detected in the parents (Figure S7). Thus, it was inferred that *BrGOLDEN* was not transformed into *B*. *rapa* by transgenic technology. Rather, it was transferred into *B*. *rapa* through distant hybridization between *B*. *rapa* and *B*. *oleracea*.

The large insertion found was a long terminal repeat (LTR) belonging to transposons, which are common in plants. Some studies have been reported in *A. thaliana* [58], *B*. *rapa* [59], and *Oryza sativa* [60], and the proportion of transposon−related sequences in each genome accounts for 10%, 39.5%, and 46%, respectively. LTRs are the most abundant transposon type in the *B*. *rapa* genome, accounting for 27.1% [59]. In animals and plants, transposons are extremely rich in functions, mainly mediating the formation of new genes, gene disruption, and regulation of gene expression or activity. The effects of transposons on genes are not only manifested at the transcriptional level but also at the post−transcriptional level [61]. These functions provide a theoretical basis into the regulation of carotenoid metabolism by *BrGOLDEN*.

The expression level of *Brgolden* in golden inner leaf Chinese cabbage was not more significant than that of *BrGOLDEN*. It is speculated that the accumulation of carotenoids is due to the new function acquired by the *BrGOLDEN* mutation, rather than the expression level. In plants, the *ORANGE* mutant exerts its functions through a variety of pathways. The differentiation of membranous chromoplasts can be induced by *AtOR^His^* or *BoOR_mut_*, but not by *AtOR* or *BoOR* [48,62]. Alternatively, *ORANGE* enhanced the accumulation and preservation of *β*−carotene and lutein in the chromoplasts, turning the tissue golden [48]. Similar results were observed in Chinese cabbage. Interestingly, in line ‘1900264′, the outer leaf tissue with high expression of the *BrGOLDEN* appeared green, similar to the control. The short stems with lower expression showed an obvious golden color, forming a ‘golden circle’ structure. Overexpression of the *PSY* encodes a key rate−limiting enzyme for carotenoid production in *A. thaliana*, but the effect of *PSY* overexpression on carotenoid accumulation in leaves is extremely small [50]. This may be because the composition and content of carotenoids are highly conserved in the green tissues, thus, limiting chromoplast formation [49]. We speculate that these two genes may limit their functions through the same pathway in green tissue.

*ORANGE* can promote membranous chromoplast formation and carotenoid accumulation in many plants [44,48,63]. For example, the overexpression of *BoOR_MUT_* in potato tubers produces orange tissue with increased carotenoid content. The mutation of a single amino acid in the *OR* (*CmOR^His^*) of melon can lead to a change in callus color and an increase in carotenoid content in *A. thaliana* and can specifically induce the formation of membranous chromoplasts [64]. In our study, *BrGOLDEN* may be derived from the cauliflower orange curd mutant but with some differences from *BoOR_MUT_*, mainly reflected in the size of the LTR fragment and SNP variation in the transcripts. *BrGOLDEN_Ins_* and *BrGOLDEN_Del_* could act alone to turn the callus golden in the induced *A. thaliana* callus and the deletion of the large fragment of *BrGOLDEN_Ldel_* limited this function. It may exert other effects by interacting with *BrPSY1* in the nucleus or acting through a *BrPSY1*−independent pathway. PSY is a vital enzyme in carotenoid synthesis and the overexpression of *AtOR* and *AtOR^His^* did not cause changes in the transcription levels of *PSY* and other enzyme genes in *A. thaliana*. Previous studies have demonstrated that *OR* may post−transcriptionally regulate the protein level of PSY [48]. This provides a reasonable explanation for the promotion of carotenoid accumulation in *B*. *rapa* by *BrGOLDEN*. Interestingly, in our study, BrGOLDEN_Del_ did not interact with BrPSY1 in the membrane system, suggesting that it may promote carotenoid accumulation through a PSY1−independent pathway. These results contribute to diversity of molecular mechanisms of carotenoid synthesis and accumulation and also provide new insight for the improvement and marker−assisted selection (MAS) of carotenoid−rich Chinese cabbage varieties.

## 4. Materials and Methods

### 4.1. Plant Materials and Callus Induction

The ‘1900264′ line, a commercial *B*. *rapa* variety, is an F_1_ cytoplasmic sterile hybrid line with golden inner leaves and a ‘golden circle’ in the short stem tissue. Line ‘1900262′ is a highly inbred *B*. *rapa* line with non−golden inner leaves and no significant carotenoid accumulation in the short stem tissue. The tri−crossed hybrid lines were obtained from the cross ‘1900264′ × ‘1900262′. All lines were cultivated in the Shunyi Base of the Chinese Academy of Agricultural Sciences. Plant samples at different stages were collected for DNA, RNA, and carotenoid analysis. The samples were frozen in liquid nitrogen for later use.

The *A. thaliana* (Col−0) was used for function verification. The *A. thaliana* seeds were plated on Murashige and Skoog (MS) medium containing 0.8% agar and 3% sucrose. The seeds were vernalized at 4 °C for 3D and then transferred to a constant temperature incubator at 22 °C with a photoperiod of 16 h light/8 h dark. After 10 d of growth, the seedlings were transplanted to the substrate until flowering.

To induce calluses, *A. thaliana* seeds were sterilized with 5% (*v*/*v*) NaClO for 15 min, washed 5–6 times with sterilized ddH_2_O and sown on seed−derived callus (SDC) medium (4.33 g/L MS basal salts/pH to 5.8 by 1 M KOH, 0.1% (*v*/*v*) Gamborg’s vitamin, 3% (*w*/*v*) sucrose, 0.5 mg/L 2,4−D, 2 mg/L indole−3−acetic acid, 0.5 mg/L 2−isopentenyladenine, 0.4% (*w*/*v*) agar) as previously described [50]. The seeds were germinated under 16 h of light/8 h of dark at 26 °C for 5 d and grown in the dark for 2 weeks in a growth incubator. Callus samples were collected, frozen in liquid nitrogen, and stored at −80 °C.

### 4.2. Genetic Analysis of the Golden Inner Leaf Phenotype

For genetic analysis, the non−golden inner leaf line ‘1900262′ (P_1_) was crossed with the golden inner leaf line ‘1900264′ (P_2_) to generate the tri−crossed hybrid lines. Phenotype characterization was performed for each generation (P_1_, P_2_, the tri−crossed hybrid lines) and the segregation ratios of the tri−crossed hybrid lines were analyzed using the chi−square (χ^2^) test. All phenotypes and samples were analyzed at the Shunyi Base, Chinese Academy of Agricultural Sciences.

### 4.3. Protoplast Separation, Microstructure Observation, and Carotenoid Analysis

In this study, the microstructure was observed by isolating protoplasts and producing free−hand slices. Protoplasts were isolated by vacuum infiltration for 30 min and gently shaking for 3 h, with 1.5 g tissue in 10 mL of a solution of 0.4 M mannitol, 1.5% (*w*/*v*) cellulose, 0.4% (*w*/*v*) macerozyme (enzymes were manufactured by Yakult Pharmaceutical Industry), 20 mM KCl, MES (pH 5.7), and 10 mM CaCl_2_. Samples were centrifuged at 100× *g* for 2 min and resuspended with 8% mannitol for observation [65]. At the mature stage, tissue sections of the inner leaves and short stems of the two parent materials were made. Sections were mounted in water under a coverslip, observed, and photographed under a light microscope (Carl Zeiss AG, Oberkochen, Germany).

The carotenoid content in the parental lines (‘1900264′ and ‘1900262′) was detected using the AB Sciex QTRAP 6500 LC−MS/MS platform. In the heading stage (14 weeks), Chinese cabbage inner leaves (~5 cm) were freeze−dried to a constant weight. The dried samples were then homogenized and powdered in a mill. The content of carotenoid components in Chinese cabbage was determined using previously described methods [66]. Three biological replicates were tested for each sample.

### 4.4. BSA−seq Mapping

The tri−crossed hybrid lines, comprising 151 individuals, were derived from a cross between lines ‘1900262′ and ‘1900264′. From the populations, equal amounts of DNA were pooled from 40 plants with a non−golden inner leaf phenotype, which constituted the non−golden pool (NGP) and from 40 plants with a golden inner leaf phenotype, which constituted the golden pool (GP). Two mixed pools and two parent pools were used for association analyses. Approximately 40× genome sequences for each pool were generated using the Illumina HiSeq 2500 platform (Illumina, San Diego, American) [67]. The *B*. *rapa* V3.0 and *A. thaliana* V10.0. were used as the reference genomes. The △SNP index and △Indel index of each position were calculated for sliding window analysis [68,69]. The SNPs and InDels were detected with GATK [70]. Gene functions were annotated with BLAST software (EMBL-EBI, Hinxton, UK) [71].

### 4.5. Cloning and Sequencing of Candidate Genes

The full−length *BrGOLDEN* sequence and CDS sequence were amplified from the gDNA and cDNA of lines ‘1900262′ and ‘1900264′ using the primers GOLDEN−F/R (Table S4). The PCR product was constructed for the T1 vector via the pEASY−T1 Cloning Kit (TransGen, Beijing, China). A single colony was selected and sent to Sangon Biotech (Sangon, Shanghai, China) for sequencing. Sequencing results were compared at http://abc.gao−lab.org/, accessed on 5 May 2021. Subsequently, transmembrane domain prediction of gene−coding proteins was performed (https://services.healthtech.dtu.dk/service.php?TMHMM-2.0, accessed on 12 May 2021). Protein structures were predicted on Phyre^2^ (http://www.sbg.bio.ic.ac.uk/phyre2/html/page.cgi?id=index, accessed on 12 May 2021). Sequence alignment of ORANGE protein sequences was performed in Cruciferae and other species. The phylogenetic tree was constructed in MEGA 7.0 software (Mega Limited, Auckland, New Zealand) and the neighbor−joining method, Poisson correction, and pairwise deletion method were used [72].

### 4.6. Quantitative Reverse−Transcription PCR

The relative expression levels of genes were analyzed via qRT−PCR. The total RNA from the seedling, rosette, and heading stages of both Chinese cabbage lines was extracted using a Plant Total RNA Mini Kit (Gene Better, Beijing, China) following the manufacturer’s instructions. The RNA quality was assessed using an RNA 6000 Nano Chip on an Agilent 2100 Bioanalyzer (http://www.chem.agilent.com, accessed on 22 April 2021). One microgram of total RNA was converted into cDNA using a reverse transcription kit (TransGen, Beijing, China). The qRT−PCR was performed using SYBR Green PCR Master Mix (Vazyme, Nanjing, China). The relative expression levels of all genes were calculated using the 2^−ΔΔCT^ method using *Bractin* as a control [73]. Three technical replicates were performed for each experiment. The primers were designed using Premier 5 (Table S4).

### 4.7. Construction of Pro_Brgolden_:GUS Fusion Vector and Histochemical Staining of Transgenic Plants

Based on the upstream sequence of *Brgolden* in the *B*. *rapa* genome database (http://brassicadb.cn, accessed on 9 September 2022), primers (Table S4) were designed to amplify the promoter sequence (~1.6 kb upstream) and ligated with the Easy−T1 vector. Prediction of promoter *cis*−acting elements was performed at Plant CARE [74]. After sequencing, the correct sequence was found by blasting in NCBI. The Pro*_Brgolden_*:GUS expression vector was constructed by restriction enzymes *Sca* I and *BamH* I and then ligated with T4 ligase (TSINGKE, Beijing, China). The recombinant vectors were then transformed into *A. thaliana Col−0* using the floral−dip method [75]. *Arabidopsis thaliana* from different tissues was selected for histochemical staining, as previously described [76].

### 4.8. Generation of Transgenic Plants Overexpressing BrGOLDEN

The complete coding region of *BrGOLDEN* was amplified from the cDNA by PCR using a pair of primers with a homologous arm (Table S4). The PCR product was inserted into the pCAMBIA1305 vector digested with *BamH* I and *Xba* I using a Trelief SoSoo Cloning Kit (TSINGKE, Beijing, China) to generate pCAMBIA1305−*BrGOLDEN_Ins_*, pCAMBIA1305−*BrGOLDEN_Ldel_*, and pCAMBIA1305−*BrGOLDEN_Del_*. The recombinant plasmid was then transferred into *E*. *coli* DH5α and sent to Sangon Biotechnology for sequencing to verify the correct insertion. We subsequently introduced the overexpression construct into *Agrobacterium tumefaciens* strain GV3101. pCAMBIA1305−*BrGOLDEN_Ins_*, pCAMBIA1305−*BrGOLDEN_Ldel_*, and pCAMBIA1305−*BrGOLDEN_Del_* were then mixed to transform *A. thaliana* [75].

### 4.9. Y2H Assay

The full−length coding sequences of *BrGOLDEN_Ldel_* and *BrPSY1* were cloned into pGBKT7 and pGADT7, respectively, following the Yeastmaker Yeast Transformation System 2 user manual (Clontech, Mountain View, CA, USA). Both plasmids were co−transformed into the golden yeast strain. The resultant strains were grown on plates for 3D at 30 °C. The interaction was tested via growth assays on media lacking leucine, tryptophan, and histidine but containing 3AT.

Interactions between BrPSY1 and BrGOLDEN_Ins_, BrGOLDEN_Ldel_, and Brgolden_wt_ were investigated in yeast with the DUAL membrane starter system (Dual−Systems Biotech). Full−length coding sequences of *BrGOLDEN_Ins_*, *BrGOLDEN_Ldel_*, and *Brgolden_wt_* were cloned into the PBT3−N vector and *BrPSY1* was cloned into the pPR3−N vector. The primers are described in Table S4. The constructed bait vector and *BrPSY1* were co−transformed into yeast strain NMY51. All strains were grown in different media: (1) SD medium lacking Trp and Leu; (2) QDO (SD medium lacking Trp, Leu, His, and Ade); and (3) QDO + 3AT (QDO with 5 mM 3−amino−1,2,4−triazole). The empty pPR3−N and PBT3 vectors were co−transformed into the NMY51 strain and tested for autoactivation with QDO medium.

### 4.10. Statistical Analysis

GraphPad Prism 8 (San Diego, CA, USA) and Microsoft Office Excel 2010 software (Redmond, WA, USA) were used to analyze the data from carotenoid content and qRT−PCR. IBM SPSS 25.0 (Armonk, NY, USA) was used to evaluate the significant differences (*p* < 0.05). Multiple *t*-tests were used for mean comparisons. The average standard deviations were adopted to indicate the measured values.

## Figures and Tables

**Table 1:** Genetic analyses of the tri−crossed hybrid populations.

Populations	Generations	Total Plants	Non−Golden Inner Leaves	Golden Inner Leaves	Expected Ratio	χ^2^ Test (Chi−Squared Test)
Tri−crossed hybrid lines	P_1_ (1900262, aa genotype)	20	20			
	P_2_ (1900264, Aa genotype)	20		20		
	P_2_ × P_1_	151	80	71	1:1	0.54

**Table 2:** Analysis of *cis*−acting elements in the *Brgolden* promoter.

Component Name	Core Sequence	Numbers	Predictive Function
MBS	CAACTG	2	MYB binding site involved in drought-inducibility
RY-element	CATGCATG	1	Cis-acting regulatory element involved in seed-specific regulation
GATA-motif	GATAGGA	1	Part of a light-responsive element
TCA-element	CCATCTTTTT	1	Cis-acting element involved in salicylic acid responsiveness
Motif I	gGTACGTGGCG	1	Cis-acting regulatory element root specific
Box 4	ATTAAT	1	Part of a conserved DNA module involved in light responsiveness
ARE	AAACCA	4	Cis-acting regulatory element essential for the anaerobic induction
ABRE	ACGTG	3	Cis-acting element involved in the abscisic acid responsiveness
TATA-box	TATATA	19	Core promoter element around −30 of transcription start
TATA-box	TATA	15	
TATA-box	TATAAAA	5	
TATA-box	TATTTAAA	1	
TATA-box	TATAA	3	
TATA-box	ccTATAAAaa	1	
TCT-motif	TCTTAC	2	Part of a light-responsive element
CAAT-box	CCAAT	9	Common cis-acting element in promoter and enhancer regions
CAAT-box	CAAT	10	
I-box	GTATAAGGCC	1	Part of a light-responsive element
G-box	CACGTG	2	Cis-acting regulatory element involved in light responsiveness
G-box	TACGTG	1	
	motif_sequence	10	Short-function
W box	TTGACC	1	Cis-acting regulatory element essential for the pathogen induction

## Data Availability

The data that support the results are included in this article and its Supplementary Materials. Other relevant materials are available from the corresponding author upon reasonable request.

## Supplementary Materials

The following supporting information can be downloaded at: https://www.mdpi.com/article/10.3390/ijms232012442/s1.
