# Supplementary material for: Mapping and Validation of BrGOLDEN: A Dominant Gene Regulating Carotenoid Accumulation in Brassica rapa
Source: Int J Mol Sci. 2022 Oct 18;23(20):12442. doi: 10.3390/ijms232012442 (PMC9603932; doi:10.3390/ijms232012442)
Supplement: Supplementary file 1 [file ijms-23-12442-s001.zip › Supplementary Figures.pdf]

Supplementary Figures

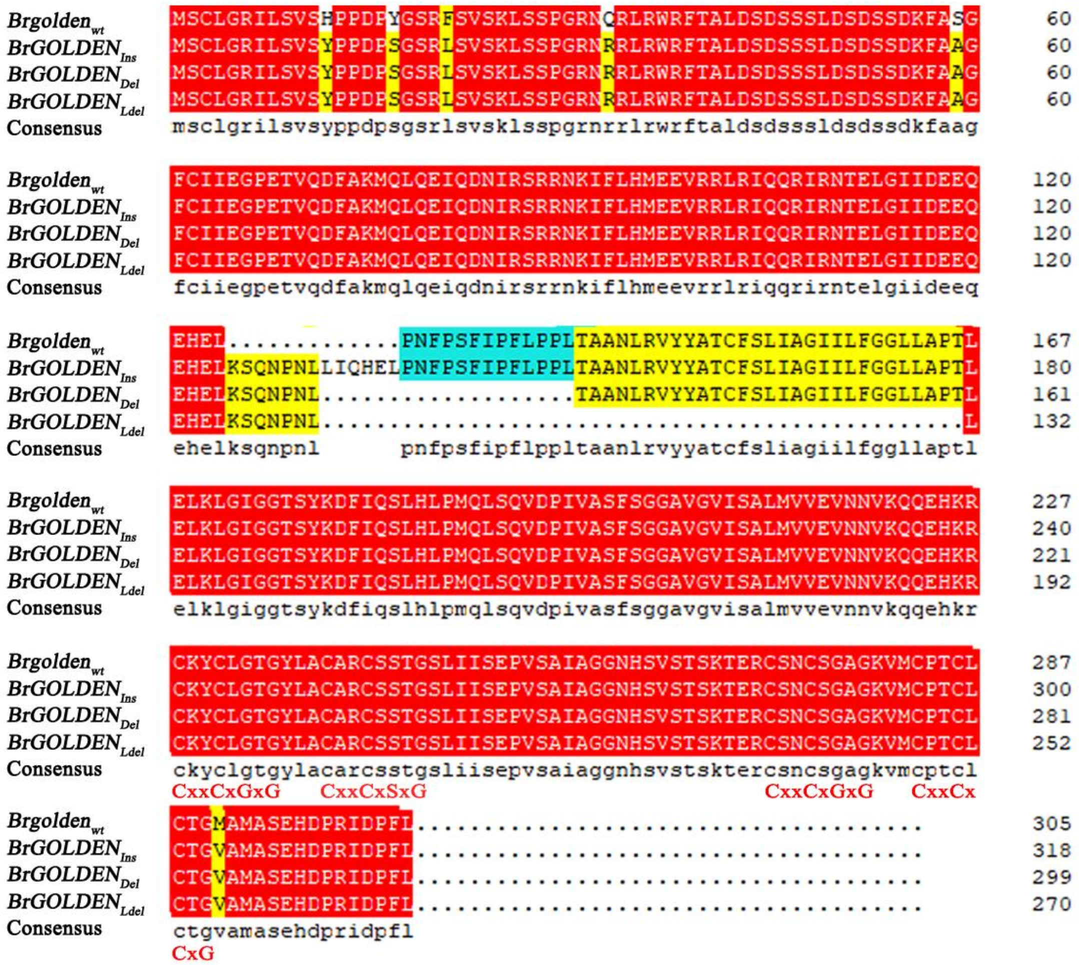

Figure S1. Comparison of amino acid sequences of five transcripts. Red words indicate four conserved C××C×××G motifs.

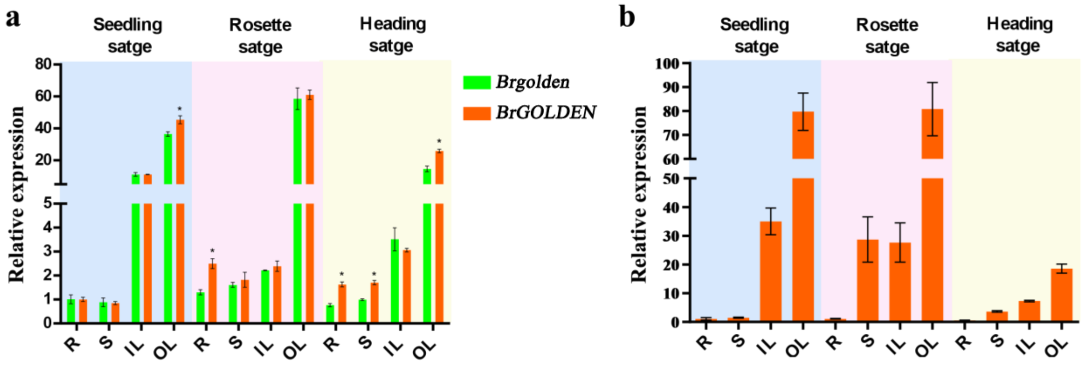

Figure S2. Comparison of *Brgolden* and *BrGOLDEN* expression in lines '1900262' and '1900264'. (**a**) Expression comparison of the *Brgolden* and *BrGOLDEN* in different periods of the line '1900264'. (**b**) Expression analysis of *Brgolden* in line '1900262'. R: roots; S: short stems; IL: inner leaves; and OL: outer leaves. Asterisk (*) indicates that there is a statistical difference, $P \leq 0.05$, $n = 3$.

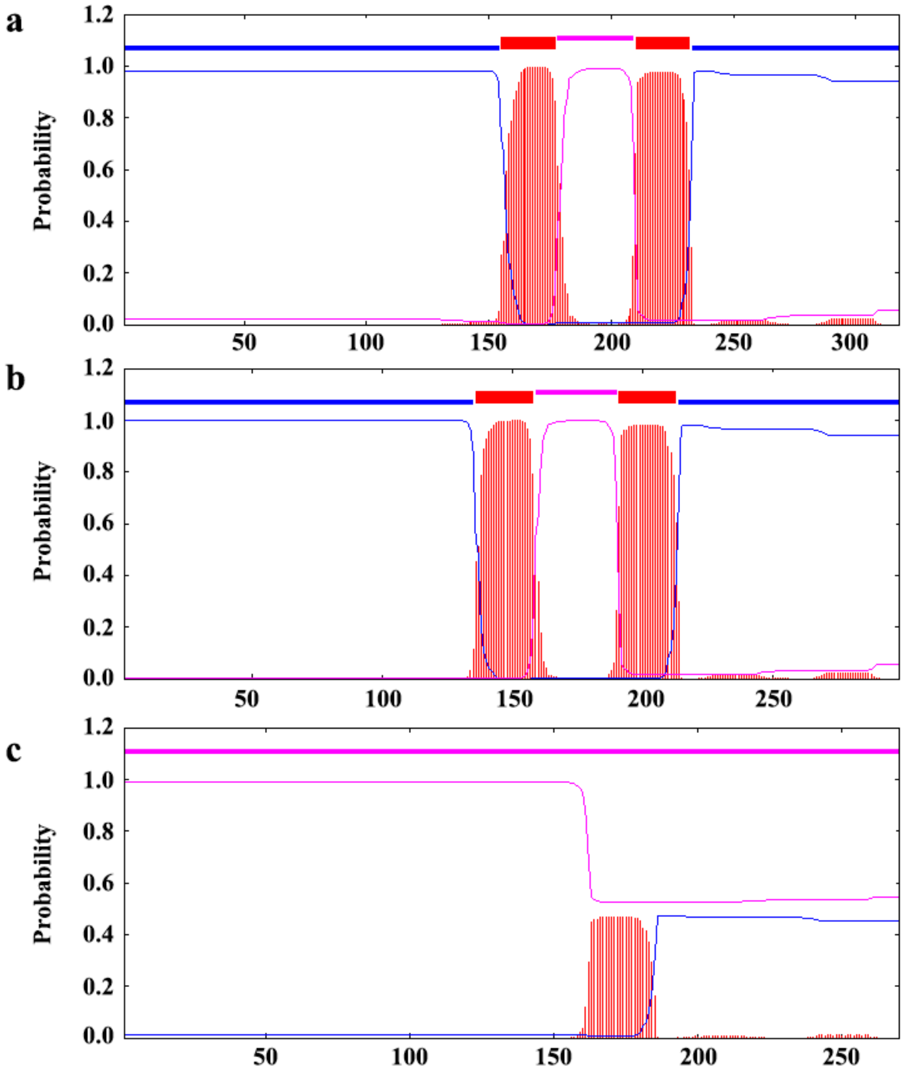

Figure S3. Prediction of transmembrane helices in BrGOLDEN proteins. Analysis of transmembrane domains of $BrGOLDEN_{Ins}$ **(a)**, $BrGOLDEN_{Del}$ (**b**), and $BrGOLDEN_{Ldel}$ (**c**). Red lines represent the transmembrane. Blue lines represent the inside. Pink lines represent the outside. The abscissa indicates the amino acid site.

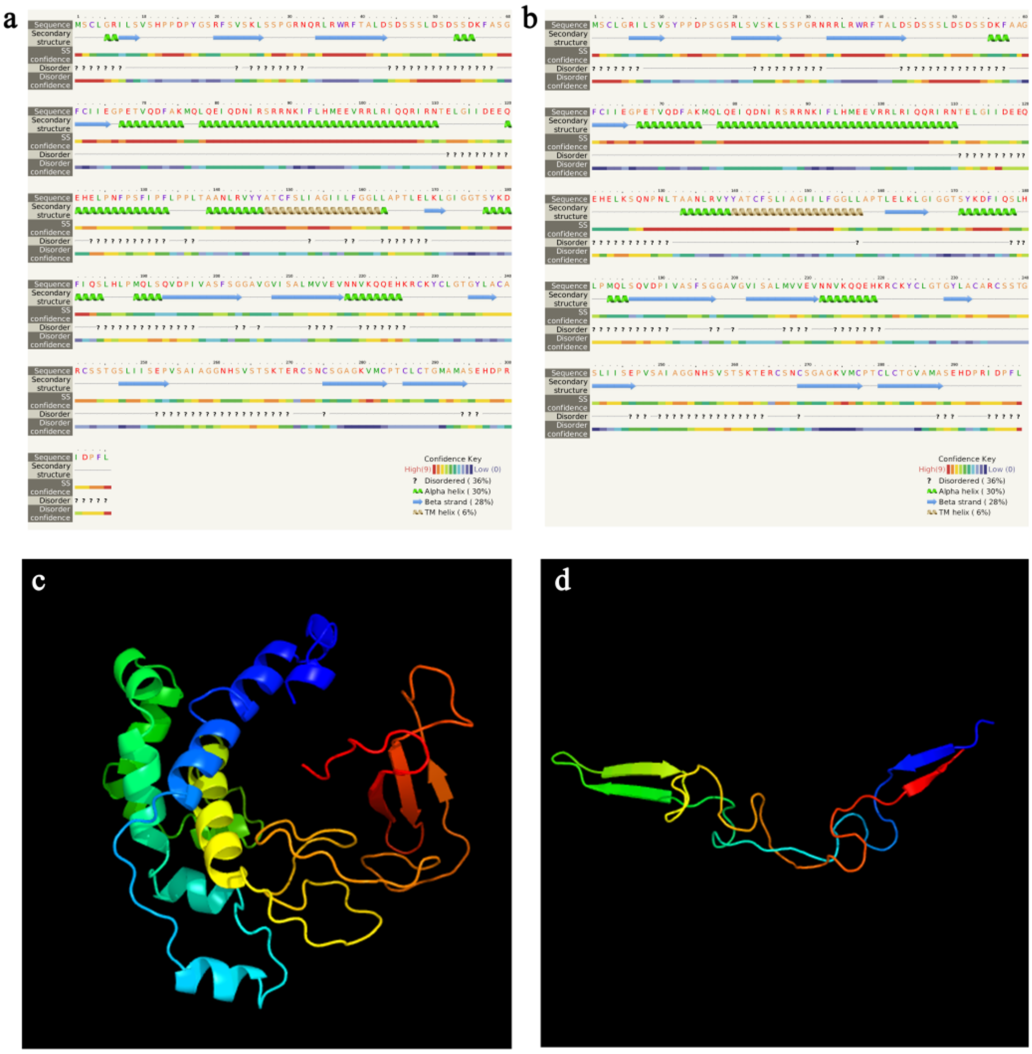

Figure S4 Phyre2 predicts protein structure. Brgolden$_{wt}$ (**a**) and BrGOLDEN$_{Del}$ (**b**) proteins structure prediction; Three-dimensional (3D) model analysis of Brgolden$_{wt}$ (**c**) and BrGOLDEN$_{Del}$ (**d**).

```
BoOR-LTR     AACACGAACTAAAGTCGCAGAATCCGAACTTGGTATGATCGTATGAACACAAACTTAATAAAACTTTTGCTTTGTCTTATTAATCTTTCTTAGCTTTCTTCTTCTTTTTTCTTATTAAAA
GOLDEN-LTR   AACACGAACTAAAGTCGCAGAATCCGAACTTGGTATGATCGTATGAACACAAACTTAATAAAACTTTTGCTTTGTCTTATTAATCTTTCTTAGCTTTCTTCTTCTTTTTTCTTATTAAAA
             ************************************************************************************************************************
BoOR-LTR     ACTCTCTTACACTTAGGCTTCTTATATAGGCAATAGAAATCTTAACTTATCCTATTACATATTAATCTAGGAATCTTTAACTTAGCTTATCTTTATCCTAAATCTATTTGTATAAGTTAT
GOLDEN-LTR   ACTCTCTTACACTTAGGCTTCTTATATAGGCAATAGAAATCTTAACTTATCCTATTACATATTAATCTAGGAATCTTTAACTTAGCTTATCTTTATCCTAAATCTATTTGTATAAGTTAT
             ************************************************************************************************************************
BoOR-LTR     TCCTCTTTCCTTTTTGAATAACTTATTACTCAAGTAACTTTGAAGCTTATCCAACATTCTCCCTCTTAAGCTTCAAACCATTTTCACTTAGGTTTTGAACTTCAATCATCGACCTCATTT
GOLDEN-LTR   TCCTCTTTCCTTTTTGAATAACTTATTACTCAAGTAACTTTGAAGCTTATCCAACATTCTCCCTCTTACTTTTCAAACCATTTTCACTTAGGTTTTGAACTTCAATCATCGACCTCATTT
             ********************************************************************   *************************************************
BoOR-LTR     CCTTGAATTTGATCTTGGCCAATGCTTTAGTTAGGATGTCTGCTTTCTGCTCTGTTCCCGGTACATGTTCCACATCAATGAGATTGCGCTCAACACATTCCCTAATGAAGTGAAACCTCT
GOLDEN-LTR   CCTTGAATTTGATCTTGGCCAATGCTTTAGTTAGGATGTCTGCTTTCTGCTCTGTTCCCGGTACATGTTCCACATCAATGAGATTGCGCTCAACACATTCCCTAATGAAGTGAAACCTCT
             ************************************************************************************************************************
BoOR-LTR     TGTGGATATGCTTGCTTCGTCGGTGAAAGACTGGATTCTTTGCAAGAGATATAGCTGACTTGTTATCGACTAGAATCAGTGTCTTCTCCGTCTCACCTCCTGTTACTTCACTCAAGAGTT
GOLDEN-LTR   TGTGGATATGCTTGCTTCGTCGGTGAAAGACTGGATTCTTTGCAAGAGATATAGCTGACTTGTTATCGACTAGAATCAGTGTCTTCTCCGTCTCACCTCCTGTTACTTCACTCAAGAGTT
             ************************************************************************************************************************
BoOR-LTR     CTTGTAACCAGATAGCCTGTTTAGCAGCCTCCGTTGCAGCCATGAACTCGGCTTCGCAGGATGACAACGCCACTGTGTCTTGTTTCTGAGAGCACCAAGTGATTGGTGTGTTTCCTAGAC
GOLDEN-LTR   CTTGTAACCAGATAGCCTGTTTAGCAGCCTCCGTTGCAGCCATGAACTCGGCTTCGCAGGATGACAACGCCACTGTGTCTTGTTTCTGAGAGCACCAAGTGATTGGTGTGTTTCCTAGAC
             ************************************************************************************************************************
BoOR-LTR     AGAACAGGTGACCTGTTGTGCTCCTTCCATCGTCCGGATCAGTATTGTGACTACTGTCGCTGTACCCTACAAGTTTCTGTGTACTTCCACGTTTAAACTCTAGACCATGCCCGAGAGTTC
GOLDEN-LTR   AGAACAGGTGACCTGTTGTGCTCCTTCCATCGTCCGGATCAGTATTGTGACTACTGTCGCTGTACCCTACAAGTTTCTGTGTACTTCCACGTTTAAACTCTAGACCATGCCCGAGAGTTC
             ************************************************************************************************************************
BoOR-LTR     CTTGTAGATATCTTAAAACTTGCTTTAACGCATTCCCGTGAGACTCTCTCGGTGATTGCATATATCTACTTAGGATCCCAACTGAGTATGCCATGTCTGGTCTAGTGTGCATTAAGTACC
GOLDEN-LTR   CTTGTAGATATCTTAAAACTTGCTTTAACGCATTCCCGTGAGACTCTCTCGGTGATTGCATATATCTACTTAGGATCCCAACTGAGTATGCCATGTCTGGTCTAGTGTGCATTAAGTACC
             ************************************************************************************************************************
BoOR-LTR     TTAAGCACCCAATCTTTCTTCGATATTGAGTTGCATCAATCTCTGGTTCATCCAGTGCCTTTGAGAATTGTAGACCGAACTCCATTGGAATACAATTTGGATTGCAATCGGCCATACCTG
GOLDEN-LTR   TTAAGCACCCAATCTTTCTTCGATATTGAGTTGCATCAATCTCTGGTTCATCCAGTGCCTTTGAGAATTGTAGACCGAACTCCATTGGAATACAATTTGGATTGCAATCGGCCATACCTG
             ************************************************************************************************************************
BoOR-LTR     ATTCTTCCATGATCCGTTTTGCATAGGCTTCTTGTTTGATTGTGATCCCTCTAGTGCTCTGCTTTACTTCAATGCCTAGATAATAGGTTAGCAACCCGAGATCAGACATTTCAAACTTGT
GOLDEN-LTR   ATTCTTCCATGATCCGTTTTGCATAGGCTTCTTGTTTGATTGTGATCCCTCTAGTGCTCTGCTTTACTTCAATGCCTAGATAATAGGTTAGCAACCCGAGATCAGACATTTCAAACTTGT
             ************************************************************************************************************************
BoOR-LTR     GTGACATCGCTGTCTTGAACTCTTTGATGATCTTTGTTGAATTTCCTGTAACAAAGAGATCATCCACATATATTGCGATAATCAGAAGCTTATCTCCTTCTTCTCTTCTGTAGACCGAGC
GOLDEN-LTR   GTGACATCGCTGTCTTGAACTCTTTGATGATCTTTGTTGAGTTTCCTGTAACAAAGAGATCATCCACATATATTGCGATAATCAGAAGCTTATCTCCTTCTTCTCTTCTGTAGACCGAGC
             **************************************** *******************************************************************************
BoOR-LTR     TTTCTTTCATGCACTTCTTGAACCGCAAACTCTTAAGAACGCGATCGAGTTTAGTATTCCAAGCGCGAGGAGCTTGCCTTAGACCATATAGTGCCTTTGAGAGTTTGAAAACTTTATGTT
GOLDEN-LTR   TTTCTTTCATGCACTTCTTGAACCGCAAACTCTTAAGAACGCGATCGAGTTTAGTATTCCAAGCGCGAGGAGCTTGCCTTAGACCATATAGTGCCTTTGAGAGTTTGAAAACTTTATGTT
             ************************************************************************************************************************
BoOR-LTR     CTTCTCCCTTTTTTTCAAACCCTTCAGGTTGAGTTACATAGACTTCTTCATTCAATTCTCCATGTAAGAATGCCGTCTTGACGTCTAGGTGGTGAAGTTCCCATCCCTTTGCAGAAGCTA
GOLDEN-LTR   CTTCTCCCTTTTTTTCAAACCCTTCAGGTTGAGTTACATAGACTTCTTCATTCAATTCTCCATGTAAGAATGCCGTCTTGACGTCTAGGTGGTGAAGTTCCCATCCCTTTGCAGAAGCTA
             ************************************************************************************************************************
BoOR-LTR     AGCTGATTAAGAGTCGAATGGACTCTATACGAGCTACTGGAGCAAACACTTCTTCAAAATCAATTCCATGCTCTTGTACATATCCCTTTGCTACAAGTCTTGCTTTGAATTTGTTGATTG
GOLDEN-LTR   AGCTGATTAAGAGTCGAATGGACTCTATACGAGCTACTGGAGCAAACACTTCTTCAAAATCAATTCCATGCTCTTGTACATATCCCTTTGCTACAAGTCTTGCTTTGAATTTGTTGATTG
             ************************************************************************************************************************
BoOR-LTR     ATCCATCTGCGTTCCTTTTGATCTTGAAAATCCACTTAAGACCAATGACTTTAACTCCGTGTGGCTTATCTACCAACTTCCATGTCTGGTTTCTGTTGATAGAGTCAATCTCATCTTCGC
GOLDEN-LTR   ATCCATCTGCGTTCCTTTTGATCTTGAAAATCCACTTAAGACCAATGACTTTAACTCCGTGTGGCTTATCTACCAACTTCCATGTCTGGTTTCTGTTGATAGAGTCAATCTCATCTTCGC
             ************************************************************************************************************************
BoOR-LTR     ATGCTTTTGTCCATCGAACATGTATCTTAGCTTCTTGGTATGTTGATGGTTCATCGTTGATTGAACACAGGAGTAGTTCGCATTCTATCTCGGCGAGTAGAATGTAATCTTCTAGGTGTT
GOLDEN-LTR   ATGCTTTTGTCCATCGAACATGTATCTTAGCTTCTTGGTATGTTGATGGTTCATCGTTGATTGAACACAGGAGTAGTTCGCATTCTATCTCGGCGAGTAGAATGTAATCTTCTAGGTGTT
             ************************************************************************************************************************
BoOR-LTR     TGGGAAGTTTTACTTCTCTAGATGATCTTCTTGGTTCGACGTGATCTACCTCGGGGGTTGGCTCTGTTGTTTCTTCTTGTTGTTCAGTCTCAGTTGCAATATTTTCTTCCTGATGTGAAC
GOLDEN-LTR   TGGGAAGTTTTACTTCTCTAGATGATCTTCTTGGTTCGACGTGATCTACCTCGGGGGTTGGCTCTGTTGTTTCTTCTTGTTGTTCAGTCTCAGTTGCAATATTTTCTTCCTGATGTGAAC
             ************************************************************************************************************************
BoOR-LTR     CTATGACAAACGGACCGTTACCCTCGTCTATGCTTGATCCCCACGTCATGTGGAACTTGCCAGATTCTTCTTGGTTTCCTTTATCTGTTTCATTCCAGTTCCAGCATGCTTTCTCATCGA
GOLDEN-LTR   CTATGACAAACGGACCGTTACCCTCGTCTATGCTTGATCCCCACGTCATGTGGAACTTGCCAGATTCTTCTTGGTTTCCTTTATCTGTTTCATTCCAGTTCCAGCATGCTTTCTCATCGA
             ************************************************************************************************************************
BoOR-LTR     ACTTCACGTCTCGACTAACCACAATCCTTCTCGTGCTAGGATTATATAAGCGGTAAGCTTTCGACCCTGGTTCTATACCAAGGTGAACCAGTGTCTGTGATCTGTCATCGAGTTTCTTCA
GOLDEN-LTR   ACTTCACGTCTCGACTAACCACAATCCTTCTCGTGCTAGGATTATATAAGCGGTAAGCTTTCGACCCTGGTTCTATACCAAGGTGAACCAGTGTCTGTGATCTGTCATCGAGTTTCTTCA
             ************************************************************************************************************************
BoOR-LTR     AAAGGGCAGCATCGAGTTTGGCATATGCCAAACAGCCAAAGACTCTGATGTGTCCAATGCTCGGCTTTCTTCCTTTGAAACTCTCATAAGGAGTTTGATTCTTCAAAGCTCTAGTTGGAA
GOLDEN-LTR   AAAGGGCAGCATCGAGTTTGGCATATGCCAAACAGCCAAAGACTCTGATGTGTCCAATGCTCGGCTTTCTTCCTTTGAAACTCTCATAAGGAGTTTGATTCTTCAAAGCTCTAGTTGGAA
             ************************************************************************************************************************
```

```
BoOR-LTR       CTCGATTGATCAGATACGTTGCATGTCGAACAGCCTCTCCCCACATATAATTTGGAACATTCATCGCTTTTAACATACTGCGCGTCATCTCCATTAACGTTCGGTTTCTTCTTTCGACAA
GOLDEN-LTR     CTCGATTGATCAGATACGTTGCATGTCGAACAGCCTCTCCCCACATATAATTTGGAACATTCATCGCTTTTAACATACTGCGCGTCATCTCCATTAACGTTCGGTTTCTTCTTTCGACAA
               ************************************************************************************************************************
BoOR-LTR       CGCCGTTTTGCTGTGGCGTATATGGCGCAGTTAGATGTCTCCTAATACCATTGTTGTTGCAGTAATCTTGAAAGTCTCTTGAAGTAAACTCTCCGCCTCTATCTGTGCGAAGTGTTACTA
GOLDEN-LTR     CGCCGTTTTGCTGTGGCGTATATGGCGCAGTTAGATGTCTCCTAATACCATTGTTGTTGCAGTAATCTTGAAAGTCTCTTGAAGTAAACTCTCCGCCTCTATCTGTGCGAAGTGTTACTA
               ************************************************************************************************************************
BoOR-LTR       TTACTTTATTGACCTCCTTCTCTACAAGAGCTTTAAAGGTCTTGAATTTCTCAAAGACTTCACTTTTTTCTTTGAGTAAAATAGACCACATGTATCTTGTGTTGTCATCAATTATGACAA
GOLDEN-LTR     TTACTTTATTGACCTCCTTCTCTACAAGAGCTTTAAAGGTCTTGAATTTCTCAAAGACTTCACTTTTTTCTTTGAGTAAAATAGACCACATGTATCTTGTGTTGTCATCAATTATGACAA
               ************************************************************************************************************************
BoOR-LTR       ATATGTACCGGTTCTGTGCCAGTGTTGAAGGAGAGATTGGACCGCAGAGGTCAGCGTGCAACAACTCTAATACCTGCGACGACCTATGTGGTGTTGCAGAGGGAAAACTATTTCGAGTCT
GOLDEN-LTR     ATATGTACCGGTTCTGTGCCAGTGTTGAAGGAGAGATTGGACCGCAGAGGTCAGCGTGCAACAACTCTAATACCTGCGACGACCTATGTGGTGTTGCAGAGGGAAAACTATTTCGAGTCT
               ************************************************************************************************************************
BoOR-LTR       GTTTCCCAACGAGACACGACTCACAAAGCTTCTTCTCTTCGTTGATCTCAGGTAGTCCTCGAACCATCTCGAGTTTTGCCATGTCTTTTATCGTCTTGAAGTTGATATGGCCAAGGCGTG
GOLDEN-LTR     GTTTCCCAACGAGACACGACTCACAAAGCTTCTTCTCTTCGTTGATCTCAGGTAGTCCTCGAACCATCTCGAGTTTTGCCATGTCTTTTATCGTCTTGAAGTTGATATGGCCAAGGCGTG
               ************************************************************************************************************************
BoOR-LTR       CGTGCCATCTCCACGGTTCTTCATTAATCTTTGTGAGAAGGCAGCTTGGTTTTCCAACTTTGAGTGATACCTTGTAGAGACGATTAGGTGATCTTAAGACTTTTACAAGTAACCTCCCGC
GOLDEN-LTR     CGTGCCATCTCCACGGTTCTTCATTAATCTTTGTGAGAAGGCAGCTTGGTTTTCCAACTTTGAGTGATACCTTGTAGAGACGATTAGGTGATCTTAAGACTTTTACAAGTAACCTCCCGC
               ************************************************************************************************************************
BoOR-LTR       TTGGATCTCTCAAAGTCAGATAGTTATCTTTCATTCTTACATCACAGCCTTGTTCAGTTGCTTGGCCTAGGCTCAGAATGTTGCTTCTTAACTCTGGTATATAATATATATTAGTTAGAA
GOLDEN-LTR     TTGGATCTCTCAAAGTCAGATAGTTATCTTTCATTCTTACATCACAGCCTTGTTCAGTTGCTTGGCCTAGGCTCAGAATGTTGCTTCTTAACTCTGGTATATAATATATATTAGTTAGAA
               ************************************************************************************************************************
BoOR-LTR       GCTTTTGCTCTCCCGTCTTTGCTTCAAAGATAATTGAGCCTTTGCCATTGATTTTCACACATGATCCATCACCAAACTTAACTCTTCCCTTGATACTTTCATTGAGTTCGGAGAAGAAGC
GOLDEN-LTR     GCTTTTGCTCTCCCGTCTTTGCTTCAAAGATAATTGAGCCTTTGCCATTGATTTTCACACATGATCCATCACCAAACTTAACTCTTCCCTTGATACTTTCATTGAGTTCGGAGAAGAAGC
               ************************************************************************************************************************
BoOR-LTR       TCTTGTCTCCTGTCATATGATTACTAGCACCGTTGTCAAGATACCAATTACCATCATCAGTTTTGTTTTGCTCAAGTTTCTTTGGCATCACGCTTTCTTCATTCAAAAACACAACTTCAT
GOLDEN-LTR     TCTTGTCTCCTGTCATATGATTACTAGCACCGTTGTCAAGATACCAATTACCATCATCAGTTTTGTTTTGCTCAAGTTTCTTTGGCATCACGCTTTCTTCATTCAAAAACACAACTTCAT
               ************************************************************************************************************************
BoOR-LTR       GCATGTATAATGCTGCTTCAGCTACTTCTGTCTCTGCTTTGTTGAGTTGATGATCATCATTCTTCTCGGGACAGACAGAGGCAAAGTGACCCTTCTTGTGGCAGTGAAAGCACTCGATTT
GOLDEN-LTR     GCATGTATAATGCTGCTTCAGCTACTTCTGTCTCTGCTTTGTTGAGTTGATGATCATCATTCTTCTCGGGACAGACAGAGGCAAAGTGACCCTTCTTGTGGCAGTGAAAGCACTCGATTT
               ************************************************************************************************************************
BoOR-LTR       GAGAGTAATCCTTCTTCTCTTTATTTCTCTCACCTCCGTTGCTACTACCACGCCCCCTTCCTCTGTTCCCACGACCCCTATTGGAACCTCGTCCTCTGCCTTTGGTTCCATGATCGGAGT
GOLDEN-LTR     GAGAGTAATCCTTCTTCTCTTTATTTCTCTCACCTCCGTTGCTACTACCACGCCCCCTTCCTCTGTTCCCACGACCCCTATTGGAACCTCGTCCTCTGCCTTTGGTTCCATGATCGGAGT
               ************************************************************************************************************************
BoOR-LTR       AGAGGAGCTTGCCATTATCTTCAAGTTTCTCTCCTCGCATTCTCTCTTCTCCACGCAAGCGTTCCTCAAATGCTTTGAGGCGACCTACCACATCTTCGTACCTTGTGTTGTTTAGGTCAA
GOLDEN-LTR     AGAGGAGCTTGCCATTATCTTCAAGTTTCTCTCCTCGCATTCTCTCTTCTCCACGCAAGCGTTCCTCAAATGCTTTGAGGCGACCTACCACATCTTCGTACCTTGTGTTGTTTAGGTCAA
               ************************************************************************************************************************
BoOR-LTR       GAACTTGTTCTAGTGTTGCTGAGAGCTGAAGAAATTTACTTGGCAGACATGATAGAAACTTCTTTACTAGTTTTGCTTCCTCTATCGTATGGCCGAGTGATGCAACTGTTCCTGCAAACT
GOLDEN-LTR     GAACTTGTTCTAGTGTTGCTGAGAGCTGAAGAAATTTACTTGGCAGACATGATAGAAACTTCTTTACTAGTTTTGCTTCCTCTATCGTATGGCCGAGTGATGCAACTGTTCCTGCAAACT
               ************************************************************************************************************************
BoOR-LTR       CTGATAATTTTCCGGAGAACGTATCTATCGAATCAGTATCTTTCATTCTCAGATGATCGAACTCAATAGACTATCCACTCAATCATCAGTGTGTGCAGGCGTGCTTCTATTACTCGATCA
GOLDEN-LTR     CTGATAATTTTCCGGAGAACGTATCTATCGAATCAGTATCTTTCATTCTCAGATGATCGA----------------ACTCAATCATCAGTGTGTGCAGGCGTGCTTCTATTACTCGATCA
               ************************************************************                ********************************************
BoOR-LTR       GCTCCAATATTCATTGCTTTCAATGCTTCCCACATACCTTTAGGAGAGTCTTGCTCCCCAACTTGTAGGATTAAGTTCTCTGGAATTCCTTGAAACAAGAGAATCGTAGCTAGGTCATTC
GOLDEN-LTR     GCTCCAATATTCATTGCTTTCAATGCTTCCCACATACCTTTAGGAGAGTCTTGCTCCCCAACTTGTAGGATTAAGTTCTCTGGAATTCCTTGAAACAAGAGAATCGTAGCTAGGTCATTC
               ************************************************************************************************************************
BoOR-LTR       TTCTTTTGGTCCTTTGATCCTGGATCAATGGTTTCCCATACCTCATGAAACCTAAATAGAACCTTCATCCTTAAAGACCAGACAGCATAGTTCGTTGTTGTGAGCATTGGTGTTTTGACA
GOLDEN-LTR     TTCTTTTGGTCCTTTGATCCTGGATCAATGGTTTCCCATACCTCATGAAACCTAAATAGAACCTTCATCCTTAAAGACCAGACAGCATAGTTCGTTGTTGTGAGCATTGGTGTTTTGACA
               ************************************************************************************************************************
BoOR-LTR       GAGGGTGGTAACGCGCTTAGCTTCATAGCTGGTTTTGTCTCTGAATCACTCATGATTAGATTAACTCATGCTCTGATACCAAATAAAGTCGCAGAATCCGAACTTGGTATGATCGTATGA
GOLDEN-LTR     GAGGGTGGTAACGCGCTTAGCTTCATAGCTGGTTTTGTCTCTGAATCACTCATGATTAGATTAACTCATGCTCTGATACCAAATAAAGTCGCAGAATCCGAACTTGGTATAATCGTATGA
               ************************************************************************************************************** *********
BoOR-LTR       ACACAAACTTAATAAAACTTTTGCTTTGTCTTATTAATCTTTCTTAGCTTTCTTCTTCTTTTTTCTTATTAAAAACTCTCTTACACTTAGGCTTCTTATATAGGCAATAGAAATCTTAAC
GOLDEN-LTR     ACACAAACTTAATAAAACTTTTGCTTTGTCTTATTAATCTTTCTTAGCTTTCTTCTTCTTTTTTCTTATTAAAAACTCTCTTACACTTAGGCTTCTTATATAGGCAATAGAAATCTTAAC
               ************************************************************************************************************************
BoOR-LTR       TTATCCTATTACATATTAATCTAGGAATCTTTAACTTAGCTTATCTTTATCCTAAATCTATTTGTATAAGTTATTCCTCTTTCCTTTTTGAATAACTTATTACTCAAGTAACTTTGAAGC
GOLDEN-LTR     TTATCCTATTACATATTAATCTAGGAATCTTTAACTTAGCTTATCTTTATCCTAAATCTATTTGTATAAGTTATTCCTCTTTCCTTTTTGAATAACTTATTACTCAAGTAACTTTGAAGC
               ************************************************************************************************************************
BoOR-LTR       TTATCC
GOLDEN-LTR     TTATCC
               ******
```

Figure S5. ***BoOR*-LTR and *BrGOLDEN*-LTR sequence comparison.** Red letters indicate different sites.

```
BoOR         ATGTCTTGTTTGGGTAGGATCTTGTCTGTTTCCTACCCACCGGATCCGTACGGTTCGCGTCTTTCAGTGTCAAAGCTGTCTTCACCAGGGAGAAACCGAAGGCTGAGATGGCGTTTCACG
GOLDEN_Ins   ATGTCTTGTTTGGGTAGGATCTTGTCTGTTTCCTACCCACCGGATCCTTCCGGTTCGCGTCTTTCAGTGTCAAAGCTGTCTTCACCAGGGAGAAACCGAAGGCTGAGATGGCGTTTCACG
GOLDEN_Del   ATGTCTTGTTTGGGTAGGATCTTGTCTGTTTCCTACCCACCGGATCCTTCCGGTTCGCGTCTTTCAGTGTCAAAGCTGTCTTCACCAGGGAGAAACCGAAGGCTGAGATGGCGTTTCACG
GOLDEN_Ldel  ATGTCTTGTTTGGGTAGGATCTTGTCTGTTTCCTACCCACCGGATCCTTCCGGTTCGCGTCTTTCAGTGTCAAAGCTGTCTTCACCAGGGAGAAACCGAAGGCTGAGATGGCGTTTCACG
             *********************************************** * **********************************************************************

BoOR         GCCTTGGACTCAGACTCTTCCTCCCTCGATTCTGATTCCTCCGACAAATTCGCTGCCGGCTTTTGTATCATAGAAGGACCTGAAACAGTACAGGACTTTGCCAAAATGCAATTACAAGAG
GOLDEN_Ins   GCCTTGGACTCAGACTCTTCCTCCCTCGATTCTGATTCCTCCGACAAATTCGCTGCCGGCTTTTGTATCATAGAAGGACCTGAAACAGTACAGGACTTTGCCAAAATGCAATTACAAGAG
GOLDEN_Del   GCCTTGGACTCAGACTCTTCCTCCCTCGATTCTGATTCCTCCGACAAATTCGCTGCCGGCTTTTGTATCATAGAAGGACCTGAAACAGTACAGGACTTTGCCAAAATGCAATTACAAGAG
GOLDEN_Ldel  GCCTTGGACTCAGACTCTTCCTCCCTCGATTCTGATTCCTCCGACAAATTCGCTGCCGGCTTTTGTATCATAGAAGGACCTGAAACAGTACAGGACTTTGCCAAAATGCAATTACAAGAG
             ************************************************************************************************************************

BoOR         ATTCAAGACAACATTAGAAGCCGTCGAAACAAGATCTTCTTGCACATGGAAGAGGTACGGAGGCTAAGAATACAACAACGGATTAGAAACACAGAGCTTGGAATCATAGACGAAGAGCAA
GOLDEN_Ins   ATTCAAGACAACATTAGAAGCCGTCGAAACAAGATCTTCTTGCACATGGAAGAGGTACGGAGGCTAAGAATACAACAACGGATTAGAAACACAGAGCTTGGAATCATAGACGAAGAGCAA
GOLDEN_Del   ATTCAAGACAACATTAGAAGCCGTCGAAACAAGATCTTCTTGCACATGGAAGAGGTACGGAGGCTAAGAATACAACAACGGATTAGAAACACAGAGCTTGGAATCATAGACGAAGAGCAA
GOLDEN_Ldel  ATTCAAGACAACATTAGAAGCCGTCGAAACAAGATCTTCTTGCACATGGAAGAGGTACGGAGGCTAAGAATACAACAACGGATTAGAAACACAGAGCTTGGAATCATAGACGAAGAGCAA
             ************************************************************************************************************************

BoOR         GAACACGAACTA---------------------------------------CCTAACTTCCCCTCCTTCATCCCATTCTTACCTCCATTGACTGCTGCCAATTTGAGAGTCTATTACGCA
GOLDEN_Ins   GAACACGAACTAAAGTCGCAGAATCCGAACTTGCTTATCCAACACGAACTACCTAACTTCCCCTCCTTCATCCCATTCTTACCTCCATTGACTGCTGCCAATTTGAGAGTCTATTACGCA
GOLDEN_Del   GAACACGAACTAAAGTCGCAGAATCCGAACTTG---------------------------------------------------------ACTGCTGCCAATTTGAGAGTCTATTACGCA
GOLDEN_Ldel  GAACACGAACTAAAGTCGCAGAATCCGAACTTG---------------------------------------------------------------------------------------
             ************

BoOR         ACTTGCTTCTCACTCATTGCTGGGATTATCCTCTTCGGTGGCCTACTAGCTCCTACTCTAGAGCTGAAGCTAGGTATAGGGGGCACATCATATAAAGATTTCATTCAAAGCCTTCATCTA
GOLDEN_Ins   ACTTGCTTCTCACTCATTGCTGGGATTATCCTCTTCGGTGGCCTACTAGCTCCTACTCTAGAGCTGAAGCTAGGTATAGGGGGCACATCATATAAAGATTTCATTCAAAGCCTTCATCTA
GOLDEN_Del   ACTTGCTTCTCACTCATTGCTGGGATTATCCTCTTCGGTGGCCTACTAGCTCCTACTCTAGAGCTGAAGCTAGGTATAGGGGGCACATCATATAAAGATTTCATTCAAAGCCTTCATCTA
GOLDEN_Ldel  ---------------------------------------------------------CTAGAGCTGAAGCTAGGTATAGGGGGCACATCATATAAAGATTTCATTCAAAGCCTTCATCTA
                                                                      ***************************************************************

BoOR         CCTATGCAATTGAGTCAAGTAGACCCAATAGTGGCGTCATTCTCTGGAGGAGCTGTTGGTGTGATCTCAGCTTTGATGGTAGTTGAAGTTAACAACGTGAAGCAGCAAGAGCACAAGAGA
GOLDEN_Ins   CCTATGCAATTGAGTCAAGTAGACCCAATAGTGGCGTCATTCTCTGGAGGAGCTGTTGGTGTGATCTCAGCTTTGATGGTAGTTGAAGTTAACAACGTGAAGCAGCAAGAGCACAAGAGA
GOLDEN_Del   CCTATGCAATTGAGTCAAGTAGACCCAATAGTGGCGTCATTCTCTGGAGGAGCTGTTGGTGTGATCTCAGCTTTGATGGTAGTTGAAGTTAACAACGTGAAGCAGCAAGAGCACAAGAGA
GOLDEN_Ldel  CCTATGCAATTGAGTCAAGTAGACCCAATAGTGGCGTCATTCTCTGGAGGAGCTGTTGGTGTGATCTCAGCTTTGATGGTAGTTGAAGTTAACAACGTGAAGCAGCAAGAGCACAAGAGA
             ************************************************************************************************************************

BoOR         TGCAAATACTGTCTAGGAACTGGGTATCTAGCATGTGCCCGTTGCTCTAGCACAGGTTCTCTTATTATATCTGAACCAGTCTCAGCTATTGCTGGAGGGAACCATTCTGTATCAACATCC
GOLDEN_Ins   TGCAAATACTGTCTAGGAACTGGGTATCTAGCATGTGCCCGTTGCTCTAGCACAGGTTCTCTTATTATATCTGAACCAGTCTCAGCTATTGCTGGAGGGAACCATTCTGTATCAACATCC
GOLDEN_Del   TGCAAATACTGTCTAGGAACTGGGTATCTAGCATGTGCCCGTTGCTCTAGCACAGGTTCTCTTATTATATCTGAACCAGTCTCAGCTATTGCTGGAGGGAACCATTCTGTATCAACATCC
GOLDEN_Ldel  TGCAAATACTGTCTAGGAACTGGGTATCTAGCATGTGCCCGTTGCTCTAGCACAGGTTCTCTTATTATATCTGAACCAGTCTCAGCTATTGCTGGAGGGAACCATTCTGTATCAACATCC
             ************************************************************************************************************************

BoOR         AAAACCGAAAGATGTTCAAACTGTTCTGGTGCTGGAAAGGTGATGTGTCCGACATGTCTGTGCACAGGAATGGCTATGGCTAGCGAGCACGACCCTCGTATTGATCCCTTCCTTTGA
GOLDEN_Ins   AAAACCGAAAGATGTTCAAACTGTTCTGGTGCTGGAAAGGTGATGTGTCCGACATGTCTGTGCACAGGAGTGGCTATGGCTAGCGAGCACGACCCTCGTATTGATCCCTTCCTTTGA
GOLDEN_Del   AAAACCGAAAGATGTTCAAACTGTTCTGGTGCTGGAAAGGTGATGTGTCCGACATGTCTGTGCACAGGAGTGGCTATGGCTAGCGAGCACGACCCTCGTATTGATCCCTTCCTTTGA
GOLDEN_Ldel  AAAACCGAAAGATGTTCAAACTGTTCTGGTGCTGGAAAGGTGATGTGTCCGACATGTCTGTGCACAGGAGTGGCTATGGCTAGCGAGCACGACCCTCGTATTGATCCCTTCCTTTGA
             ********************************************************************* ***********************************************
```

Figure S6. Three transcripts of the *BrGOLDEN* and open reading frames of *BoOR* sequence comparison. Red letters indicated different sites.

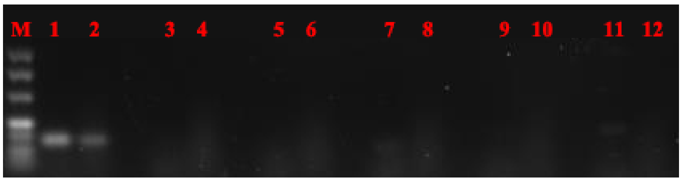

Figure S7. RT-PCR detection of specific regulatory elements. M: Marker 500 bp; The specific expressions of primers *Bractin* (lanes 1 and 2), *CaMV* 35S promoter (lanes 3 and 4), *FMV* 35S promoter (lanes 5 and 6), *NOS* promoter (lanes 7 and 8), *NOS* terminator (lanes 9 and 10) and *CaMV* 35S terminator (lanes 11 and 12) were detected in the '1900264' and '1900262' lines.
