# Supplementary material for: Mapping and Validation of BrGOLDEN: A Dominant Gene Regulating Carotenoid Accumulation in Brassica rapa
Source: Int J Mol Sci. 2022 Oct 18;23(20):12442. doi: 10.3390/ijms232012442 (PMC9603932; doi:10.3390/ijms232012442)
Supplement: Supplementary file 1 [file ijms-23-12442-s001.zip › Supplementary Tables.pdf]

Supplementary Tables

Table S1 Qualitative and quantitative detection of carotenoids in Chinese cabbage

Compounds	1900262-1	1900262-2	1900262-3	1900264-1	1900264-2	1900264-3	Pvalue (1900262_vs_1900264)
β-Carotene	13.4	18.4	11.7	119	108	93.8	0.003833015
α-Carotene	0.253	0.317	0.364	1.62	1.17	1.36	0.010893401
Lutein	61.5	96.6	53.3	201	142	170	0.011060411
Neoxanthin	3.93	5.44	3.14	8.75	4.49	5.25	0.269787026
β-Cryptoxanthin	1.27	1.01	0.93	4.47	2.53	3.42	0.04630673
Antheraxanthin	0.214	0.262	0.162	0.624	0.271	0.502	0.12574915
Violaxanthin	2.3	2.52	1.89	4.19	2.31	2.9	0.242896814
(E/Z)-Phytoene	N/A	N/A	N/A	16.7	16.2	12.8	N/A
lutein laurate	1.04	1.1	0.928	1.2	1.04	1.1	0.257707013
β-cryptoxanthin laurate	0.737	0.573	0.501	1.35	0.573	0.471	0.560242992
violaxanthin myristate	0.0597	0.0518	0.0312	0.07	0.0425	0.0462	0.682158456
lutein palmitate	1.34	1.7	1.3	2.31	2.31	2.27	0.020708166
lutein dimyristate	0.965	0.976	0.911	1.19	1.05	1.03	0.094411035
lutein dilaurate	1.03	1.14	0.928	1.22	1.17	1.22	0.099267502
violaxanthin palmitate	0.0519	0.107	0.033	0.111	0.122	0.129	0.118321896
lutein myristate	0.975	1.1	0.934	1.16	0.992	1.16	0.25031158
Capsorubin	0.202	0.179	0.348	0.0934	N/A	N/A	N/A
Lycopene	N/A	N/A	N/A	N/A	N/A	N/A	N/A
Zeaxanthin	N/A	N/A	N/A	N/A	N/A	N/A	N/A

γ-Carotene	N/A	N/A	N/A	N/A	N/A	N/A	N/A
Astaxanthin	N/A	N/A	N/A	N/A	N/A	N/A	N/A
Apocarote--l	N/A	N/A	N/A	N/A	N/A	N/A	N/A
Phytofluene	N/A	N/A	N/A	N/A	N/A	N/A	N/A
Capsanthin	N/A	N/A	N/A	N/A	N/A	N/A	N/A
ε-Carotene	N/A	N/A	N/A	N/A	N/A	N/A	N/A
α-Cryptoxanthin	N/A	N/A	N/A	N/A	N/A	N/A	N/A
antheraxanthin dipalmitate	N/A	N/A	N/A	N/A	N/A	N/A	N/A
lutein caprate	N/A	N/A	N/A	N/A	N/A	N/A	N/A
lutein stearate	N/A	N/A	N/A	N/A	N/A	N/A	N/A
5,6epoxy-luttein dilaurate	N/A	N/A	N/A	N/A	N/A	N/A	N/A
5,6epoxy-lutein-caprate-palmitate	N/A	N/A	N/A	N/A	N/A	N/A	N/A
lutein dipalmitate	N/A	0.933	N/A	1.13	0.998	0.992	N/A
lutein distearate	N/A	N/A	N/A	N/A	N/A	N/A	N/A
lutein dioleate	N/A	N/A	N/A	0.924	N/A	N/A	N/A
lutein oleate	N/A	N/A	N/A	N/A	N/A	N/A	N/A
neochrome palmitate	N/A	N/A	N/A	N/A	N/A	N/A	N/A
rubixanthin caprate	N/A	N/A	N/A	N/A	N/A	N/A	N/A
rubixanthin laurate	N/A	N/A	N/A	N/A	N/A	N/A	N/A
rubixanthin myristate	N/A	N/A	N/A	N/A	N/A	N/A	N/A
rubixanthin palmitate	N/A	N/A	N/A	N/A	N/A	N/A	N/A
violaxanthin dibutyrate	N/A	N/A	N/A	N/A	N/A	N/A	N/A
violaxanthin laurate	N/A	N/A	N/A	N/A	N/A	N/A	N/A
violaxanthin palmitoleate	N/A	N/A	N/A	N/A	N/A	N/A	N/A
violaxanthin dilaurate	N/A	N/A	N/A	N/A	N/A	N/A	N/A
violaxanthin-myristate-caprate	N/A	N/A	N/A	N/A	N/A	N/A	N/A

violaxanthin-myristate-laurate	N/A	N/A	N/A	N/A	N/A	N/A	N/A
violaxanthin dimyristate	N/A	N/A	N/A	N/A	N/A	N/A	N/A
violaxanthin-myristate-palmitate	N/A	N/A	N/A	N/A	N/A	N/A	N/A
violaxanthin dipalmitate	N/A	N/A	N/A	N/A	N/A	N/A	N/A
violaxanthin-myristate-oleate	N/A	N/A	N/A	N/A	N/A	N/A	N/A
violaxanthin dioleate	N/A	N/A	N/A	N/A	N/A	N/A	N/A
zeaxanthin myristoleate	N/A	N/A	N/A	N/A	N/A	N/A	N/A
zeaxanthin palmitate	N/A	N/A	N/A	N/A	N/A	N/A	N/A
zeaxanthin-caprate-laurate	N/A	N/A	N/A	N/A	N/A	N/A	N/A
zeaxanthin dilaurate	N/A	N/A	N/A	N/A	N/A	N/A	N/A
zeaxanthin-laurate-myristate	N/A	N/A	N/A	N/A	N/A	N/A	N/A
zeaxanthin dimyristate	N/A	N/A	N/A	N/A	N/A	N/A	N/A
zeaxanthin-laurate-palmitate	N/A	N/A	N/A	N/A	N/A	N/A	N/A
zeaxanthin-myristate-palmitate	N/A	N/A	N/A	N/A	N/A	N/A	N/A
zeaxanthin dipalmitate	N/A	N/A	N/A	N/A	N/A	N/A	N/A
zeaxanthin-palmitate-stearate	N/A	N/A	N/A	N/A	N/A	N/A	N/A
zeaxanthin-oleate-palmitate	N/A	N/A	N/A	N/A	N/A	N/A	N/A
β-cryptoxanthin myristate	N/A	N/A	N/A	N/A	N/A	N/A	N/A
β-cryptoxanthin oleate	N/A	N/A	N/A	N/A	N/A	N/A	N/A
canthaxanthin	N/A	N/A	N/A	N/A	N/A	N/A	N/A
echinenone	N/A	N/A	N/A	0.00634	N/A	0.00444	N/A
β-citraurin	N/A	N/A	N/A	N/A	N/A	N/A	N/A
β-cryptoxanthin palmitate	0.438	0.496	N/A	0.926	1.19	0.742	N/A

Table S2 BSA-seq raw data

Generations		Clean Reads	Clean Bases	Q30 Percentage (%)	GC Percentage (%)	Mapped (%)	Properly mapped (%)	Average depth	Coverage ratio 1X (%)	Coverage ratio 5X (%)	Coverage ratio 10X (%)
P_1 (1900262, *aa* genotype)		60,064,028	17,964,551,756	92.46	37.72	98.08	95.34	34	94.61	92.60	91.06
P_2 (1900264, *Aa genotype*）		71,027,935	17,964,551,756	92.97	37.61	98.16	95.12	41	97.00	95.56	94.28
BC_1	NGP	90,277,996	17,964,551,756	93.33	37.62	98.41	95.91	48	97.93	96.79	95.50
	GP	89,932,917	26,905,462,562	92.69	37.36	98.39	95.06	48	98.03	96.90	95.65

Table S3. All gene information in the candidate region

Gene_ID	Pfam_annotation	Swissprot_annotation
BraA09g003880.3C	NB-ARC domain;; NLI interacting factor-like phosphatase	Probable disease resistance protein At1g59620
BraA09g006110.3C	Sigma 54 modulation/S30EA ribosomal protein C terminus;; Sigma 54 modulation protein / S30EA ribosomal protein	Ribome-binding factor PSRP1, chloroplastic
BraA09g007180.3C	--	--
BraA09g009100.3C	--	Protein TOC75-4, chloroplastic
BraA09g003900.3C	KIX domain	Probable mediator of RNA polymerase II transcription subunit 15c
BraA09g009200.3C	Domain of unknown function (DUF4283);; Zinc knuckle	--
BraA09g008480.3C	Ring finger domain;; zinc-ribbon;; Zinc finger, C3HC4 type (RING finger);; RING-like zinc finger;; Zinc finger, C3HC4 type (RING finger)	E3 ubiquitin-protein ligase CIP8
BraA09g005500.3C	--	--
BraA09g005230.3C	C2H2-type zinc finger	Zinc finger protein 4
BraA09g007190.3C	--	--
BraA09g008450.3C	Ras of Complex, Roc, domain of DAPkinase;; Ras family	Small GTPase LIP1
BraA09g005210.3C	Ribosomal protein 60S L18 and 50S L18e;; Ribosomal proteins 50S-L15, 50S-L18e, 60S-L27A	60S ribomal protein L18-2
BraA09g008700.3C	--	--
BraA09g008470.3C	4-alpha-glucanotransferase	4-alpha-glucanotransferase DPE1, chloroplastic/amyloplastic
BraA09g006480.3C	Sugar efflux transporter for intercellular exchange	Bidirectional sugar transporter SWEET12
BraA09g008260.3C	Rad51	DNA repair protein XRCC2 homolog
BraA09g005490.3C	tRNA synthetases class I (E and Q), catalytic domain;; tRNA synthetases class I (E and Q), anti-codon binding domain;; Glutathione S-transferase, C-terminal domain;; Glutathione S-transferase, C-terminal domain	Glutamate--tRNA ligase, cytoplasmic
BraA09g010030.3C	Histone-like transcription factor (CBF/NF-Y) and archaeal histone;; Core histone H2A/H2B/H3/H4	Nuclear transcription factor Y subunit B-7
BraA09g009810.3C	Rhodanese-like domain	Thiulfate sulfurtransferase 16, chloroplastic
BraA09g005440.3C	PurA ssDNA and RNA-binding protein	Transcription factor Pur-alpha 1
BraA09g008370.3C	Ribosomal proteins 50S-L15, 50S-L18e, 60S-L27A	--
BraA09g005150.3C	Rapid ALkalinization Factor (RALF)	Protein RALF-like 33
BraA09g009170.3C	Helix-loop-helix DNA-binding domain	Transcription factor HEC1

BraA09g008630.3C	Cyclophilin type peptidyl-prolyl cis-trans isomerase/CLD	Peptidyl-prolyl cis-trans isomerase CYP38, chloroplastic
BraA09g008170.3C	Alternative oxidase	Ubiquinol oxidase 2, mitochondrial
BraA09g006530.3C	Ribosomal proteins 50S L24/mitochondrial 39S L24;; KOW motif	50S ribomal protein L24, chloroplastic
BraA09g005310.3C	Drought induced 19 protein (Di19), zinc-binding;; Stress-induced protein Di19, C-terminal	Protein DEHYDRATION-INDUCED 19 homolog 6
BraA09g009060.3C	WD domain, G-beta repeat;; Anaphase-promoting complex subunit 4 WD40 domain;; Eukaryotic translation initiation factor eIF2A;; LisH	WD40 repeat-containing protein H15
BraA09g006210.3C	Dipeptidyl peptidase IV (DPP IV) N-terminal region;; Prolyl oligopeptidase family;; X-Pro dipeptidyl-peptidase (S15 family)	Probable glutamyl endopeptidase, chloroplastic
BraA09g006880.3C	NYN domain;; Zinc-finger of C2H2 type;; Zinc-finger double-stranded RNA-binding	--
BraA09g009890.3C	Protein tyrosine kinase;; Protein kinase domain	Calmodulin-binding receptor-like cytoplasmic kinase 3
BraA09g005240.3C	--	Zinc finger protein 3
BraA09g007440.3C	Ras family;; Ras of Complex, Roc, domain of DAPkinase;; ADP-ribosylation factor family	Rac-like GTP-binding protein ARAC10
BraA09g008130.3C	--	--
BraA09g007360.3C	POT family	Protein NRT1/ PTR FAMILY 2.11
BraA09g006800.3C	Nuclear transport factor 2 (NTF2) domain;; RNA recognition motif. (a.k.a. RRM, RBD, or RNP domain)	Nuclear transport factor 2
BraA09g009380.3C	--	--
BraA09g007450.3C	TFIIS helical bundle-like domain;; BAH domain	--
BraA09g006660.3C	TCP family transcription factor	Transcription factor TCP7
BraA09g005850.3C	BAH domain	Protein ANTI-SILENCING 1
BraA09g005050.3C	--	Uncharacterized protein At4g28440
BraA09g009720.3C	Oxidoreductase NAD-binding domain	Ferredoxin--NADP reductase, leaf isozyme 1, chloroplastic
BraA09g008460.3C	Cleavage site for pathogenic type III effector avirulence factor Avr	--
BraA09g010020.3C	Malic enzyme, NAD binding domain;; Malic enzyme, N-terminal domain	NAD-dependent malic enzyme 1, mitochondrial
BraA09g004930.3C	Domain of unknown function (DUF3444);; DnaJ domain	Chaperone protein dnaJ 49
BraA09g007240.3C	Microtubule associated protein (MAP65/ASE1 family)	65-kDa microtubule-associated protein 9
BraA09g007660.3C	--	--
BraA09g007470.3C	--	--
BraA09g009340.3C	SAP domain	Zinc finger CCCH domain-containing protein 62

BraA09g009710.3C	Protein kinase domain;; EF hand;; Protein tyrosine kinase;; EF hand;; EF-hand domain;; EF-hand domain pair;; EF-hand domain pair;; Secreted protein acidic and rich in cysteine Ca binding region	Calcium-dependent protein kinase 28
BraA09g008100.3C	--	--
BraA09g007790.3C	Bromodomain;; Bromodomain extra-terminal - transcription regulation	Transcription factor GTE10
BraA09g008310.3C	Glycosyl hydrolase family 3 C-terminal domain;; Glycosyl hydrolase family 3 N terminal domain;; Fibronectin type III-like domain	Beta-D-xylidase 4
BraA09g005720.3C	--	--
BraA09g007780.3C	Nucleoside diphosphate kinase	Nucleide diphphate kinase II, chloroplastic
BraA09g008000.3C	Regulator of chromosome condensation (RCC1) repeat;; Regulator of chromosome condensation (RCC1) repeat	Ultraviolet-B receptor UVR8
BraA09g008140.3C	cAMP-regulated phosphoprotein/endosulfine conserved region	--
BraA09g004990.3C	Amino-transferase class IV	Branched-chain-amino-acid aminotransferase-like protein 2
BraA09g006490.3C	Protein kinase domain;; EF hand;; Protein tyrosine kinase;; EF-hand domain;; EF-hand domain pair;; EF-hand domain pair;; EF hand;; EF-hand domain;; Cytoskeletal-regulatory complex EF hand;; Secreted protein acidic and rich in cysteine Ca binding region	Calcium-dependent protein kinase 12
BraA09g006680.3C	Protein of unknown function, DUF617	Protein MIZU-KUSSEI 1
BraA09g009070.3C	Cytochrome P450;; EB1-like C-terminal motif;; Calponin homology (CH) domain	Cytochrome P450 81D1
BraA09g004940.3C	--	--
BraA09g005680.3C	16S rRNA methyltransferase RsmB/F;; Methyltransferase domain;; ubiE/COQ5 methyltransferase family	--
BraA09g008510.3C	Acyl-CoA oxidase;; Acyl-CoA dehydrogenase, middle domain;; Acyl-CoA dehydrogenase, C-terminal domain	Acyl-coenzyme A oxidase 2, peroxisomal
BraA09g006380.3C	Ribosomal protein L13e	60S ribomal protein L13-2
BraA09g010080.3C	Peroxidase	Peroxidase 32
BraA09g005040.3C	EF hand associated;; Ras family;; EF hand associated;; Seryl-tRNA synthetase N-terminal domain;; Ras of Complex, Roc, domain of DAPkinase;; 50S ribosome-binding GTPase;; EF-hand domain	Mitochondrial Rho GTPase 1
BraA09g005640.3C	Alfin;; PHD-finger	PHD finger protein ALFIN-LIKE 3
BraA09g008570.3C	Seed dormancy control;; bZIP transcription factor;; Basic region leucine zipper	Transcription factor TGA1
BraA09g008990.3C	--	--

BraA09g007530.3C	RNA polymerase Rpb4	--
BraA09g005350.3C	Calmodulin binding protein-like	Calmodulin-binding protein 60 G
BraA09g009490.3C	Protein DA1;; LIM domain	Protein DA1-related 3
BraA09g006620.3C	GRAM domain	Putative GEM-like protein 8
BraA09g009740.3C	Rad17 cell cycle checkpoint protein;; ATPase family associated with various cellular activities (AAA)	Cell cycle checkpoint protein RAD17
BraA09g007510.3C	Domain of unknown function (DUF4228)	--
BraA09g005420.3C	U1 zinc finger;; Zinc finger C-x8-C-x5-C-x3-H type (and similar)	Zinc finger CCCH domain-containing protein 3
BraA09g005760.3C	Protein of unknown function (DUF1677)	--
BraA09g008860.3C	Domain of unknown function (DUF4228)	--
BraA09g008120.3C	--	--
BraA09g005290.3C	--	--
BraA09g006630.3C	Iron/manganese superoxide dismutases, C-terminal domain;; Iron/manganese superoxide dismutases, alpha-hairpin domain	Superoxide dismutase [Fe] 3, chloroplastic
BraA09g009290.3C	Multicopper oxidase;; Multicopper oxidase;; Multicopper oxidase	L-ascorbate oxidase homolog
BraA09g008650.3C	Cyclin, N-terminal domain;; Cyclin, C-terminal domain	Cyclin-D4-1
BraA09g005450.3C	--	--
BraA09g008210.3C	3,4-dihydroxy-2-butanone 4-phosphate synthase;; GTP cyclohydrolase II	Bifunctional riboflavin biynthesis protein RIBA 1, chloroplastic
BraA09g006930.3C	Response regulator receiver domain;; CCT motif	Two-component response regulator-like APRR1
BraA09g004960.3C	Domain of unknown function (DUF4283);; Zinc knuckle	Uncharacterized protein At4g02000
BraA09g007050.3C	AP2 domain	Ethylene-responsive transcription factor ERF107
BraA09g003890.3C	KIX domain	Probable mediator of RNA polymerase II transcription subunit 15c
BraA09g005580.3C	Plastocyanin-like domain	Mavicyanin
BraA09g009660.3C	Peroxidase	Peroxidase 72
BraA09g009260.3C	--	Molybdenum cofactor sulfurase
BraA09g007010.3C	RhoGAP domain	Uncharacterized Rho GTPase-activating protein At5g61530
BraA09g010070.3C	DHHC palmitoyltransferase;; Ankyrin repeats (3 copies);; Ankyrin repeats (many copies);; Ankyrin repeat;; Ankyrin repeat;; Ankyrin repeats (many copies)	Probable protein S-acyltransferase 23
BraA09g006320.3C	Transferase family	BAHD acyltransferase At5g47980
BraA09g010140.3C	--	--
BraA09g010000.3C	Aminotransferase class-V	Serine--glyoxylate aminotransferase

BraA09g009210.3C	Lipase (class 3);; Protein of unknown function (DUF2974)	Phpholipase A1-Igamma2, chloroplastic
BraA09g007020.3C	Protein kinase domain;; Protein tyrosine kinase;; U-box domain;; Universal stress protein family	U-box domain-containing protein 52
BraA09g008660.3C	14-3-3 protein	14-3-3-like protein GF14 kappa
BraA09g007990.3C	Glycosyl hydrolases family 31;; Galactose mutarotase-like	Probable glucan 1,3-alpha-glucidase
BraA09g003830.3C	--	Protein RIK
BraA09g005610.3C	MATH domain	Ubiquitin carboxyl-terminal hydrolase 12
BraA09g008090.3C	Transferase family	Uncharacterized acetyltransferase At3g50280
BraA09g005030.3C	PPR repeat;; PPR repeat family;; Pentatricopeptide repeat domain	Pentatricopeptide repeat-containing protein At5g27460
BraA09g007430.3C	Sugar efflux transporter for intercellular exchange	Bidirectional sugar transporter SWEET5
BraA09g007850.3C	--	Mediator of RNA polymerase II transcription subunit 30
BraA09g005970.3C	WD domain, G-beta repeat;; WD40 associated region in TFIID subunit, NTD2 domain;; Anaphase-promoting complex subunit 4 WD40 domain	Transcription initiation factor TFIID subunit 5
BraA09g006450.3C	Ribosomal_S17 N-terminal;; Ribosomal protein S17	40S ribomal protein S11-3
BraA09g008880.3C	linker histone H1 and H5 family;; Myb-like DNA-binding domain;; Myb-like DNA-binding domain	Telomere repeat-binding factor 2
BraA09g006780.3C	COBRA-like protein	Protein COBRA
BraA09g005460.3C	Myb-like DNA-binding domain;; Myb-like DNA-binding domain	Transcription factor MYB86
BraA09g007280.3C	No apical meristem (NAM) protein	NAC domain-containing protein 101
BraA09g009310.3C	--	Probable disease resistance protein At5g66890
BraA09g008940.3C	--	--
BraA09g006440.3C	Remorin, C-terminal region;; Remorin, N-terminal region	Remorin
BraA09g003910.3C	Cytochrome P450	Cytochrome P450 85A2
BraA09g007860.3C	CBS domain;; PB1 domain	CBS domain-containing protein CBSCBSPB1
BraA09g005530.3C	Adenylate kinase;; AAA domain;; AAA domain;; AAA domain;; Thymidylate kinase	UMP-CMP kinase 3
BraA09g008600.3C	Protein of unknown function, DUF617	Protein MIZU-KUSSEI 1
BraA09g005810.3C	Ubiquitin-conjugating enzyme	Protein PEROXIN-4
BraA09g007670.3C	Histone deacetylase domain	Histone deacetylase 6
BraA09g008180.3C	CG-1 domain;; Ankyrin repeats (3 copies);; IQ calmodulin-binding motif;; Ankyrin repeats (many copies)	Calmodulin-binding transcription activator 2
BraA09g005300.3C	Kinesin motor domain;; Microtubule binding;; Calponin homology (CH) domain	Kinesin-like protein KIN-14G

BraA09g006560.3C	con80 domain of Katanin	Katanin p80 WD40 repeat-containing subunit B1 homolog
BraA09g005670.3C	Papain family cysteine protease	Thiol protease aleurain
BraA09g005200.3C	Folate receptor family	--
BraA09g006350.3C	C2 domain	--
BraA09g007580.3C	--	--
BraA09g007130.3C	APO RNA-binding	APO protein 3, mitochondrial
BraA09g009800.3C	Bifunctional nuclease;; UvrB/uvrC motif	Bifunctional nuclease 2
BraA09g005840.3C	BURP domain	BURP domain protein RD22
BraA09g009630.3C	GATA zinc finger	GATA transcription factor 5
BraA09g006890.3C	Ankyrin repeats (3 copies);; Ankyrin repeats (many copies);; Ankyrin repeat;; Ankyrin repeat;; Ankyrin repeats (many copies)	Phytochrome-interacting ankyrin-repeat protein 2
BraA09g008380.3C	--	--
BraA09g008320.3C	GDP-fucose protein O-fucosyltransferase	O-fucyltransferase 38
BraA09g007680.3C	DEAD/DEAH box helicase;; Helicase conserved C-terminal domain	DEAD-box ATP-dependent RNA helicase 30
BraA09g009580.3C	Cellulase (glycosyl hydrolase family 5)	Mannan endo-1,4-beta-mannidase 7
BraA09g007390.3C	HPP family	--
BraA09g005380.3C	tRNA synthetase class II core domain (G, H, P, S and T);; Anticodon binding domain;; TGS domain;; Threonyl and Alanyl tRNA synthetase second additional domain	Threonine--tRNA ligase, mitochondrial 1
BraA09g006870.3C	Ribosomal protein S19e	40S ribomal protein S19-3
BraA09g005100.3C	Domain of unknown function (DUF1995)	--
BraA09g005590.3C	MATH domain	Ubiquitin carboxyl-terminal hydrolase 12
BraA09g008050.3C	SRF-type transcription factor (DNA-binding and dimerisation domain)	Agamous-like MADS-box protein AGL61
BraA09g005600.3C	MATH domain	Ubiquitin carboxyl-terminal hydrolase 12
BraA09g008410.3C	Cellulose synthase;; Zinc-binding RING-finger;; Glycosyl transferase family group 2	Cellule synthase A catalytic subunit 6 [UDP-forming]
BraA09g005070.3C	Mitochondrial carrier protein	Peroxisomal adenine nucleotide carrier 2
BraA09g008720.3C	Dof domain, zinc finger	Dof zinc finger protein DOF5.7
BraA09g005260.3C	--	--
BraA09g009650.3C	Mitochondrial carrier protein	Folate transporter 1, chloroplastic
BraA09g008540.3C	Aluminium induced protein	--
BraA09g007000.3C	Zinc-binding dehydrogenase;; Zinc-binding dehydrogenase;; Alcohol	--

	dehydrogenase GroES-like domain	
BraA09g009240.3C	B3 DNA binding domain	Putative B3 domain-containing protein At5g66980
BraA09g006770.3C	K-box region;; SRF-type transcription factor (DNA-binding and dimerisation domain)	Agamous-like MADS-box protein AGL8
BraA09g005430.3C	G-patch domain;; Zinc-finger double-stranded RNA-binding;; G-patch domain	Septin and tuftelin-interacting protein 1 homolog 1
BraA09g008290.3C	--	Probable WRKY transcription factor 19
BraA09g006100.3C	60s Acidic ribosomal protein	60S acidic ribomal protein P1
BraA09g005800.3C	Tesmin/TSO1-like CXC domain, cysteine-rich domain;; Alpha/beta hydrolase family;; Acetyl xylan esterase (AXE1);; Prolyl oligopeptidase family;; Dienelactone hydrolase family	Protein tesmin/TSO1-like CXC 7
BraA09g007720.3C	Anaphase-promoting complex subunit 15	--
BraA09g006950.3C	--	--
BraA09g005920.3C	Chloroplast import apparatus Tic20-like	Protein TIC 20-II, chloroplastic
BraA09g004980.3C	--	Branched-chain-amino-acid aminotransferase-like protein 1
BraA09g005880.3C	AP2 domain	Ethylene-responsive transcription factor SHINE 3
BraA09g008420.3C	--	Ethylene-responsive transcription factor ABR1
BraA09g007890.3C	RecQ mediated genome instability protein;; Recq-mediated genome instability protein 1, C-terminal OB-fold	RecQ-mediated genome instability protein 1
BraA09g008340.3C	Plant invertase/pectin methylesterase inhibitor	Cell wall / vacuolar inhibitor of fructidase 2
BraA09g008080.3C	Hyccin	--
BraA09g007540.3C	--	--
BraA09g007830.3C	Cytochrome P450	Cytochrome P450 94B1
BraA09g005130.3C	C-terminus of histone H2A;; Core histone H2A/H2B/H3/H4	Probable histone H2A.5
BraA09g009350.3C	--	Precursor of CEP7
BraA09g007380.3C	POT family	Protein NRT1/ PTR FAMILY 2.11
BraA09g006190.3C	Glycosyl hydrolases family 17;; X8 domain	Glucan endo-1,3-beta-glucidase
BraA09g006370.3C	Kinesin motor domain;; Microtubule binding;; Helix-hairpin-helix motif	Kinesin-like protein KIN-10C
BraA09g008070.3C	Photosystem I reaction centre subunit N (PSAN or PSI-N)	Photystem I reaction center subunit N, chloroplastic
BraA09g006290.3C	--	--
BraA09g006710.3C	bZIP transcription factor;; Basic region leucine zipper;; bZIP Maf transcription factor	Basic leucine zipper 43
BraA09g006510.3C	Got1/Sft2-like family	--

BraA09g009280.3C	Autophagy-related protein 101	Autophagy-related protein 101
BraA09g008010.3C	Calcineurin-like phosphoesterase	Serine/threonine-protein phphatase 7
BraA09g008800.3C	Aminotransferase class I and II	1-aminocyclopropane-1-carboxylate synthase 5
BraA09g010120.3C	NPH3 family;; BTB/POZ domain	BTB/POZ domain-containing protein NPY2
BraA09g008280.3C	No apical meristem (NAM) protein	NAC domain-containing protein 104
BraA09g009610.3C	--	--
BraA09g007750.3C	Tetratricopeptide repeat;; Tetratricopeptide repeat;; Tetratricopeptide repeat;; Tetratricopeptide repeat;; Tetratricopeptide repeat;; TPR repeat;; Tetratricopeptide repeat;; Tetratricopeptide repeat;; Tetratricopeptide repeat;; Tetratricopeptide repeat;; Tetratricopeptide repeat;; Tetratricopeptide repeat	Probable UDP-N-acetylglucamine--peptide N-acetylglucaminyltransferase SEC
BraA09g008620.3C	MatE	Protein DETOXIFICATION 27
BraA09g005400.3C	--	Nuclear envelope-associated protein 2
BraA09g006750.3C	Protein kinase domain;; Protein tyrosine kinase;; D-mannose binding lectin;; PAN-like domain	G-type lectin S-receptor-like serine/threonine-protein kinase RLK1
BraA09g009430.3C	--	--
BraA09g007090.3C	Inositol polyphosphate kinase	Initol polyphphate multikinase beta
BraA09g005090.3C	Kinesin motor domain;; Microtubule binding	Kinesin-like protein KIN-14S
BraA09g007400.3C	Protein of unknown function (DUF1645)	--
BraA09g007730.3C	Calcineurin-like phosphoesterase	Probable inactive purple acid phphatase 29
BraA09g008440.3C	WRKY DNA -binding domain	Probable WRKY transcription factor 51
BraA09g005250.3C	--	--
BraA09g008500.3C	Nucleotide-sugar transporter;; Purine nucleobase transmembrane transport	CMP-sialic acid transporter 5
BraA09g005820.3C	PPR repeat family;; PPR repeat;; PPR repeat;; Pentatricopeptide repeat domain;; Pentacotripeptide-repeat region of PRORP;; Tetratricopeptide repeat;; Tetratricopeptide repeat;; Tetratricopeptide repeat;; Evolutionarily conserved signalling intermediate in Toll pathway	Pentatricopeptide repeat-containing protein At5g25630
BraA09g005830.3C	Flavin-binding monooxygenase-like;; Pyridine nucleotide-disulphide oxidoreductase;; L-lysine 6-monooxygenase (NADPH-requiring);; Pyridine nucleotide-disulphide oxidoreductase;; FAD binding domain;; NAD(P)-binding Rossmann-like domain;; Pyridine nucleotide-disulphide oxidoreductase	Indole-3-pyruvate monooxygenase YUCCA6
BraA09g009400.3C	SNF2 family N-terminal domain;; U1 small nuclear ribonucleoprotein of 70kDa MW N terminal;; Helicase conserved C-terminal domain;; RNA recognition motif. (a.k.a. RRM, RBD, or RNP domain);; Type III restriction enzyme, res	ATP-dependent DNA helicase DDM1

	subunit	
BraA09g007120.3C	Domain of unknown function DUF220	--
BraA09g007550.3C	Plant organelle RNA recognition domain	Protein WHAT≈S THIS FACTOR 1 homolog
BraA09g010130.3C	Zn-finger in ubiquitin-hydrolases and other protein	--
BraA09g009940.3C	--	--
BraA09g010110.3C	Helix-loop-helix DNA-binding domain	Transcription factor bHLH84
BraA09g007210.3C	DEAD/DEAH box helicase;; GUCT (NUC152) domain;; Helicase conserved C-terminal domain;; Type III restriction enzyme, res subunit	DEAD-box ATP-dependent RNA helicase 7
BraA09g003860.3C	Transferase family	BAHD acyltransferase At3g29680
BraA09g005470.3C	Pectinacetylesterase	Pectin acetylesterase 10
BraA09g009640.3C	Glutaredoxin	Glutaredoxin-C3
BraA09g009040.3C	--	--
BraA09g006900.3C	Leucine Rich repeats (2 copies);; Leucine rich repeat;; Leucine Rich Repeat;; Leucine Rich repeat;; Leucine rich repeat N-terminal domain	Leucine-rich repeat receptor protein kinase MSP1
BraA09g005930.3C	--	Hydroxyproline O-arabinyltransferase 1
BraA09g007910.3C	Aminotransferase class-III	Glutamate-1-semialdehyde 2,1-aminomutase, chloroplastic
BraA09g009860.3C	Kinesin motor domain;; Microtubule binding;; MyTH4 domain;; FERM central domain;; FERM N-terminal domain	Kinesin-like protein KIN-14E
BraA09g007900.3C	DEK C terminal domain	--
BraA09g008810.3C	--	Uncharacterized protein At3g49720
BraA09g006590.3C	Leucine rich repeat;; Leucine Rich repeats (2 copies);; Leucine Rich Repeat;; Leucine rich repeat N-terminal domain;; Leucine Rich repeat	Leucine-rich repeat receptor-like protein kinase PXC2
BraA09g009390.3C	FAD binding domain;; Fumarate reductase flavoprotein C-term	Succinate dehydrogenase [ubiquinone] flavoprotein subunit 1, mitochondrial
BraA09g008360.3C	Ribosomal protein L17	50S ribomal protein L17, chloroplastic
BraA09g009770.3C	2OG-Fe(II) oxygenase superfamily;; 2OG-Fe(II) oxygenase superfamily	Probable prolyl 4-hydroxylase 10
BraA09g007630.3C	Ribosomal protein S19	40S ribomal protein S15-6
BraA09g006240.3C	Sigma-70 region 3;; Sigma-70, region 4;; Sigma-70 region 2	RNA polymerase sigma factor sigE, chloroplastic/mitochondrial
BraA09g008610.3C	Core histone H2A/H2B/H3/H4	Histone H3.2
BraA09g009790.3C	--	--
BraA09g007330.3C	Helix-loop-helix DNA-binding domain	Transcription factor bHLH79

BraA09g008790.3C	Myb-like DNA-binding domain;; Myb-like DNA-binding domain	Transcription factor RAX3
BraA09g006570.3C	--	GPI-anchored protein LLG1
BraA09g006000.3C	Protein of unknown function (DUF1336)	Protein ENHANCED DISEASE RESISTANCE 2-like
BraA09g007030.3C	Protein kinase domain;; Protein tyrosine kinase;; Universal stress protein family	U-box domain-containing protein 52
BraA09g009870.3C	Hydrophobic seed protein;; Probable lipid transfer	pEARLI1-like lipid transfer protein 1
BraA09g008870.3C	ETC complex I subunit conserved region	NADH dehydrogenase [ubiquinone] iron-sulfur protein 4, mitochondrial
BraA09g005360.3C	Domain of unknown function (DUF4378);; DUF761-associated sequence motif	--
BraA09g007480.3C	MuDR family transposase;; SWIM zinc finger	--
BraA09g005510.3C	DEAD/DEAH box helicase;; Helicase conserved C-terminal domain;; GUCT (NUC152) domain;; Type III restriction enzyme, res subunit;; Zinc knuckle	DEAD-box ATP-dependent RNA helicase 3, chloroplastic
BraA09g006810.3C	Replication factor-A C terminal domain;; Replication factor-A protein 1, N-terminal domain;; Replication protein A OB domain;; OB-fold nucleic acid binding domain;; Domain of unknown function DUF223	Replication protein A 70 kDa DNA-binding subunit D
BraA09g008770.3C	Aminotransferase class-V;; Beta-eliminating lyase;; DegT/DnrJ/EryC1/StrS aminotransferase family	Cysteine desulfurase, mitochondrial
BraA09g009950.3C	Serine carboxypeptidase	Serine carboxypeptidase-like 42
BraA09g005020.3C	GHMP kinases N terminal domain;; GHMP kinases C terminal	Mevalonate kinase
BraA09g006140.3C	RNA recognition motif. (a.k.a. RRM, RBD, or RNP domain)	Polyadenylate-binding protein-interacting protein 13
BraA09g007250.3C	Domain of unknown function (DUF296)	AT-hook motif nuclear-localized protein 6
BraA09g006600.3C	--	Probable gluce-1-phphate adenylyltransferase large subunit, chloroplastic
BraA09g006090.3C	No apical meristem (NAM) protein	NAC domain-containing protein 91
BraA09g004950.3C	Eukaryotic glutathione synthase, ATP binding domain;; Eukaryotic glutathione synthase	Glutathione synthetase, chloroplastic
BraA09g007950.3C	Pyruvate kinase, barrel domain;; Pyruvate kinase, alpha/beta domain	Pyruvate kinase, cytolic isozyme
BraA09g005340.3C	GATA zinc finger	GATA transcription factor 23
BraA09g007500.3C	Permease family	Nucleobase-ascorbate transporter 6
BraA09g009510.3C	Cysteine-rich secretory protein family	STS14 protein
BraA09g007820.3C	FBD	FBD-associated F-box protein At4g10400
BraA09g006500.3C	Cleft lip and palate transmembrane protein 1 (CLPTM1);; PQ loop repeat	--
BraA09g006150.3C	RNA recognition motif. (a.k.a. RRM, RBD, or RNP domain)	Polyadenylate-binding protein-interacting protein 13

BraA09g007940.3C	Alcohol dehydrogenase GroES-like domain	Probable mannitol dehydrogenase
BraA09g007140.3C	Auxin response factor;; B3 DNA binding domain;; AUX/IAA family	Auxin response factor 2
BraA09g008960.3C	Lateral organ boundaries (LOB) domain	LOB domain-containing protein 37
BraA09g005790.3C	AP2 domain	Ethylene-responsive transcription factor TINY
BraA09g006220.3C	Phosphatidylinositol 3- and 4-kinase	Phphatidylinitol 4-kinase gamma 3
BraA09g003820.3C	--	--
BraA09g009690.3C	--	--
BraA09g005550.3C	Pyruvate phosphate dikinase, PEP/pyruvate binding domain;; Starch binding domain	Phphoglucan, water dikinase, chloroplastic
BraA09g005160.3C	--	--
BraA09g005660.3C	--	--
BraA09g006390.3C	S-layer homology domain	--
BraA09g003920.3C	Myb-like DNA-binding domain;; Myb-like DNA-binding domain	Transcription factor MYB62
BraA09g008640.3C	ZF-HD protein dimerisation region	Zinc-finger homeodomain protein 1
BraA09g006940.3C	Exonuclease	Protein NEN2
BraA09g008690.3C	Protein tyrosine kinase;; U-box domain;; Protein kinase domain	Putative U-box domain-containing protein 50
BraA09g009090.3C	Dihydrouridine synthase (Dus);; Histidine biosynthesis protein;; Dihydroorotate dehydrogenase	tRNA-dihydrouridine(47) synthase [NAD(P)(+)]-like
BraA09g007700.3C	Taurine catabolism dioxygenase TauD, TfdA family	Clavaminate synthase-like protein At3g21360
BraA09g005140.3C	Ribosomal protein S21e	40S ribomal protein S21-2
BraA09g009550.3C	Domain of unknown function DUF223;; Replication protein A OB domain	--
BraA09g006160.3C	RNA recognition motif. (a.k.a. RRM, RBD, or RNP domain)	Polyadenylate-binding protein-interacting protein 13
BraA09g008300.3C	CorA-like Mg2+ transporter protein	Magnesium transporter MRS2-2
BraA09g008020.3C	Histidinol dehydrogenase	Histidinol dehydrogenase, chloroplastic
BraA09g009920.3C	Monogalactosyldiacylglycerol (MGDG) synthase;; Glycosyltransferase family 28 C-terminal domain	Monogalactyldiacylglycerol synthase 3, chloroplastic
BraA09g009440.3C	Homeobox domain;; Homeobox KN domain	Homeobox-leucine zipper protein ATHB-53
BraA09g006610.3C	Protein of unknown function (DUF789)	--
BraA09g005900.3C	--	--
BraA09g009530.3C	Manganese-stabilising protein / photosystem II polypeptide	Oxygen-evolving enhancer protein 1-1, chloroplastic
BraA09g006200.3C	EF hand;; EF-hand domain;; EF-hand domain pair;; EF hand;; EF-hand domain pair	Calcineurin B-like protein 4

BraA09g008930.3C	C2H2-type zinc finger;; C2H2-type zinc finger;; Zinc-finger double-stranded RNA-binding	Zinc finger protein AZF1
BraA09g009700.3C	--	--
BraA09g009970.3C	Glutathione S-transferase, N-terminal domain;; Glutathione S-transferase, C-terminal domain;; Glutathione S-transferase, N-terminal domain;; Glutathione S-transferase, N-terminal domain;; Glutathione S-transferase, C-terminal domain	Glutathione S-transferase F14
BraA09g006020.3C	--	--
BraA09g006360.3C	--	--
BraA09g006050.3C	--	tRNA (guanine-N(7)-)-methyltransferase
BraA09g009150.3C	Transferase family	Protein ENHANCED PSEUDOMONAS SUSCEPTIBILTY 1
BraA09g006330.3C	Transferase family	BAHD acyltransferase At5g47980
BraA09g010010.3C	SNF2 family N-terminal domain;; Chromo (CHRromatin Organisation MOdifier) domain;; Helicase conserved C-terminal domain;; Domain of unknown function (DUF4208);; Class II histone deacetylase complex subunits 2 and 3;; Type III restriction enzyme, res subunit	Protein CHROMATIN REMODELING 5
BraA09g008040.3C	Small VCP/p97-interacting protein	--
BraA09g006260.3C	Protein kinase domain;; Protein tyrosine kinase;; Leucine rich repeat;; Leucine Rich repeats (2 copies);; Leucine rich repeat N-terminal domain	Inactive leucine-rich repeat receptor-like serine/threonine-protein kinase At5g24100
BraA09g006030.3C	--	Flowering-promoting factor 1
BraA09g007080.3C	--	Protein ORANGE, chloroplastic
BraA09g008970.3C	Transcriptional regulator of RNA polII, SAGA, subunit	--
BraA09g006230.3C	Squalene epoxidase;; FAD binding domain;; FAD dependent oxidoreductase;; FAD binding domain;; NAD(P)-binding Rossmann-like domain;; Glucose inhibited division protein A	Squalene monooxygenase 1,1
BraA09g005560.3C	--	T-complex protein 1 subunit gamma
BraA09g008710.3C	UDP-glucoronosyl and UDP-glucosyl transferase	UDP-glycyltransferase 91B1
BraA09g009560.3C	--	--
BraA09g008760.3C	Protein kinase domain;; Protein tyrosine kinase;; Leucine rich repeat;; Leucine Rich repeats (2 copies);; Leucine Rich Repeat;; Leucine rich repeat N-terminal domain	LRR receptor-like serine/threonine-protein kinase HSL2
BraA09g009000.3C	Protein of unknown function (DUF1230)	Protein CONSERVED IN THE GREEN LINEAGE AND DIATOMS 27, chloroplastic
BraA09g006690.3C	Histone-like transcription factor (CBF/NF-Y) and archaeal histone	Protein Dr1 homolog

BraA09g003850.3C	Transferase family	Malonyl-CoA:anthocyanidin 5-O-glucide-6≈≈-O-malonyltransferase
BraA09g007230.3C	Cell cycle regulated microtubule associated protein	--
BraA09g006740.3C	Myb-like DNA-binding domain;; Myb-like DNA-binding domain	Transcription factor MYB34
BraA09g006060.3C	Coatomer WD associated region;; WD domain, G-beta repeat	--
BraA09g007970.3C	--	Protein KOKOPELLI
BraA09g009570.3C	50S ribosome-binding GTPase;; KH domain;; Ferrous iron transport protein B;; RsgA GTPase;; Dynamin family;; Conserved hypothetical ATP binding protein	GTPase ERA-like, chloroplastic
BraA09g005190.3C	Ribosomal L18 of archaea, bacteria, mitoch. and chloroplast	--
BraA09g009360.3C	--	Precursor of CEP6
BraA09g009590.3C	PPR repeat family;; PPR repeat;; PPR repeat;; Pentatricopeptide repeat domain;; Pentacotripeptide-repeat region of PRORP;; Tetratricopeptide repeat;; Tetratricopeptide repeat;; Tetratricopeptide repeat;; Tetratricopeptide repeat;; Tetratricopeptide repeat	Putative pentatricopeptide repeat-containing protein At3g47840
BraA09g009220.3C	FAD binding domain;; FHA domain;; NAD(P)-binding Rossmann-like domain;; FAD binding domain	Zeaxanthin epoxidase, chloroplastic
BraA09g009250.3C	Prolyl oligopeptidase, N-terminal beta-propeller domain;; Prolyl oligopeptidase family;; Secretory lipase	--
BraA09g009830.3C	RNA recognition motif. (a.k.a. RRM, RBD, or RNP domain)	--
BraA09g009230.3C	RWP-RK domain	Protein RKD3
BraA09g006250.3C	WRKY DNA -binding domain	Probable WRKY transcription factor 30
BraA09g009420.3C	Protein tyrosine kinase;; Protein kinase domain;; Kinase-like	Serine/threonine-protein kinase HT1
BraA09g006860.3C	Leo1-like protein	Protein LEO1 homolog
BraA09g009520.3C	Domain of unknown function (DUF4228)	--
BraA09g008190.3C	Splicing factor 3B subunit 1;; HEAT repeat;; HEAT repeats;; HEAT-like repeat;; non-SMC mitotic condensation complex subunit 1;; Vacuolar 14 Fab1-binding region	--
BraA09g007110.3C	Calreticulin family	Calnexin homolog 1
BraA09g006790.3C	COBRA-like protein	COBRA-like protein 5
BraA09g005390.3C	IGR protein motif	--
BraA09g008670.3C	Domain of unknown function (DUF4283);; Zinc knuckle	Uncharacterized protein At4g02000
BraA09g005170.3C	LSM domain	Sm-like protein LSM4

BraA09g009270.3C	Dof domain, zinc finger	Dof zinc finger protein DOF5.8
BraA09g007150.3C	Hsp70 protein;; MreB/Mbl protein;; NAD-specific glutamate dehydrogenase	Probable mediator of RNA polymerase II transcription subunit 37e
BraA09g007520.3C	Dof domain, zinc finger	Dof zinc finger protein DOF5.6
BraA09g005940.3C	--	--
BraA09g008740.3C	AUX/IAA family	Auxin-responsive protein IAA9
BraA09g007070.3C	--	Spliceome-associated protein 130 B
BraA09g006840.3C	Histone deacetylase domain	Histone deacetylase 5
BraA09g007260.3C	Protein of unknown function (DUF1442)	--
BraA09g005630.3C	ChaC-like protein	Gamma-glutamylcyclotransferase 2-1
BraA09g008590.3C	Protein of unknown function (DUF740)	--
BraA09g007170.3C	SRF-type transcription factor (DNA-binding and dimerisation domain);; K-box region	MADS-box protein AGL42
BraA09g008820.3C	Leucine rich repeat;; Leucine Rich repeats (2 copies);; Leucine Rich Repeat	Inactive LRR receptor-like serine/threonine-protein kinase BIR2
BraA09g008270.3C	--	--
BraA09g006340.3C	--	--
BraA09g009370.3C	--	--
BraA09g006470.3C	RNA polymerase Rpc34 subunit	--
BraA09g008730.3C	--	Uncharacterized protein At5g65660
BraA09g007560.3C	Glutaredoxin	Glutaredoxin-C1
BraA09g008550.3C	--	--
BraA09g005650.3C	Mitochondrial carrier protein	Folate transporter 1, chloroplastic
BraA09g009820.3C	SacI homology domain	Phphoinitide phphatase SAC6
BraA09g004970.3C	Tim44-like domain	--
BraA09g006550.3C	--	--
BraA09g006120.3C	Response regulator receiver domain;; CCT motif	Two-component response regulator-like APRR5
BraA09g006720.3C	Dof domain, zinc finger	Dof zinc finger protein DOF5.4
BraA09g009990.3C	C2 domain	BON1-associated protein 2
BraA09g009030.3C	Subtilase family;; Peptidase inhibitor I9;; PA domain	Subtilisin-like protease SBT1.7
BraA09g007960.3C	SWIB/MDM2 domain;; Plus-3 domain	Uncharacterized protein At5g08430
BraA09g007290.3C	Myb-like DNA-binding domain;; Myb-like DNA-binding domain	Transcription factor MYB94

BraA09g006910.3C	Apoptosis antagonizing transcription factor;; Apoptosis-antagonizing transcription factor, C-terminal	--
BraA09g007640.3C	Cupin-like domain;; JmjC domain, hydroxylase	F-box protein At1g78280
BraA09g007880.3C	Heavy-metal-associated domain	Heavy metal-associated isoprenylated plant protein 7
BraA09g008230.3C	FKBP-type peptidyl-prolyl cis-trans isomerase	Peptidyl-prolyl cis-trans isomerase FKBP12
BraA09g009110.3C	NB-ARC domain	Disease resistance protein RPP13
BraA09g009670.3C	Dehydrin	Dehydrin Rab18
BraA09g009330.3C	Ribosomal protein TL5, C-terminal domain	--
BraA09g007460.3C	Domain of unknown function (DUF1985);; Ulp1 protease family, C-terminal catalytic domain	--
BraA09g005540.3C	Response regulator receiver domain	Two-component response regulator ARR22
BraA09g010060.3C	Aldehyde dehydrogenase family	Methylmalonate-semialdehyde dehydrogenase [acylating], mitochondrial
BraA09g007270.3C	Plant invertase/pectin methylesterase inhibitor	21 kDa protein
BraA09g006540.3C	alpha/beta hydrolase fold;; Carboxylesterase family	Probable carboxylesterase 18
BraA09g005180.3C	Ribosomal L22e protein family	60S ribomal protein L22-2
BraA09g007650.3C	Lateral organ boundaries (LOB) domain	Protein LATERAL ORGAN BOUNDARIES
BraA09g009980.3C	Glycosyltransferase family 17	--
BraA09g008850.3C	--	--
BraA09g007690.3C	Translin family	Mitochondrial import inner membrane translocase subunit TIM14-3
BraA09g007420.3C	Peroxisomal membrane anchor protein (Pex14p) conserved region	Peroxisomal membrane protein PEX14
BraA09g008980.3C	Peroxidase	Peroxidase 73
BraA09g005890.3C	Leucine Rich repeat;; F-box domain;; F-box-like;; Leucine Rich Repeat	EIN3-binding F-box protein 2
BraA09g006920.3C	Protein tyrosine kinase;; Protein kinase domain;; Carbohydrate-binding protein of the ER;; Di-glucose binding within endoplasmic reticulum	Probable receptor-like protein kinase At5g61350
BraA09g007760.3C	Protein of unknown function (DUF410)	--
BraA09g006980.3C	Myb-like DNA-binding domain;; Myb-like DNA-binding domain	Transcription factor MYB28
BraA09g005570.3C	Sugar (and other) transporter;; Major Facilitator Superfamily	Sugar transport protein 13
BraA09g006180.3C	Glucosamine-6-phosphate isomerases/6-phosphogluconolactonase	Probable 6-phphogluconolactonase 5, chloroplastic
BraA09g008890.3C	ADP-ribosylation factor family;; Ras family;; Ras of Complex, Roc, domain of DAPkinase;; Gtr1/RagA G protein conserved region;; Signal recognition particle	ADP-ribylation factor-like protein 8b

	receptor beta subunit;; 50S ribosome-binding GTPase	
BraA09g005520.3C	--	--
BraA09g009190.3C	--	--
BraA09g007160.3C	--	--
BraA09g006760.3C	D-mannose binding lectin	G-type lectin S-receptor-like serine/threonine-protein kinase RLK1
BraA09g008920.3C	Eukaryotic porin	Mitochondrial outer membrane protein porin 2
BraA09g009080.3C	--	Probable beta-1,4-xylyltransferase IRX14H
BraA09g008900.3C	--	--
BraA09g005730.3C	Plant self-incompatibility protein S1	--
BraA09g008030.3C	PHD-finger;; Acetyltransferase (GNAT) domain;; Acetyltransferase (GNAT) family	Increased DNA methylation 1
BraA09g005780.3C	Exostosin family	Probable glycyltransferase At5g03795
BraA09g008400.3C	Prolamin-like	Egg cell-secreted protein 1.5
BraA09g006640.3C	Dihydroorotate dehydrogenase	Dihydroorotate dehydrogenase (quinone), mitochondrial
BraA09g007590.3C	--	--
BraA09g007200.3C	DUF761-associated sequence motif	--
BraA09g008430.3C	Glycosyl hydrolases family 17;; X8 domain	Glucan endo-1,3-beta-glucidase 8
BraA09g009140.3C	OTU-like cysteine protease;; SEC-C motif	--
BraA09g009780.3C	Ankyrin repeats (3 copies);; Ankyrin repeats (many copies);; Ankyrin repeats (many copies);; Ankyrin repeat;; Ankyrin repeat	Ankyrin repeat domain-containing protein, chloroplastic
BraA09g005000.3C	--	--
BraA09g007610.3C	--	--
BraA09g009880.3C	Zinc knuckle	--
BraA09g007920.3C	non-haem dioxygenase in morphine synthesis N-terminal;; 2OG-Fe(II) oxygenase superfamily	Flavonol synthase/flavanone 3-hydroxylase
BraA09g007810.3C	Protein tyrosine kinase;; Protein kinase domain;; Leucine rich repeat;; Leucine rich repeat N-terminal domain	Probable inactive leucine-rich repeat receptor-like protein kinase At3g03770
BraA09g009450.3C	UDP-glucoronosyl and UDP-glucosyl transferase	UDP-glycyltransferase 72E3
BraA09g007740.3C	Pectate lyase;; Right handed beta helix region	Probable pectate lyase 22
BraA09g008840.3C	U-box domain	U-box domain-containing protein 31
BraA09g009850.3C	Alpha and gamma adaptin binding protein p34;; Ras family	--

BraA09g006850.3C	X8 domain	PLASMODESMATA CALLE-BINDING PROTEIN 1
BraA09g006430.3C	--	Heavy metal-associated isoprenylated plant protein 39
BraA09g007710.3C	PB1 domain	--
BraA09g006970.3C	--	--
BraA09g010040.3C	Sodium/hydrogen exchanger family	Cation/H(+) antiporter 15
BraA09g007320.3C	Exportin 1-like protein	Transportin M14
BraA09g008680.3C	--	--
BraA09g008520.3C	--	--
BraA09g003870.3C	Transferase family	BAHD acyltransferase At3g29680
BraA09g007620.3C	--	--
BraA09g007370.3C	--	Protein NRT1/ PTR FAMILY 2.11
BraA09g007490.3C	TFIIS helical bundle-like domain;; BAH domain	--
BraA09g005410.3C	Protein kinase domain;; Protein tyrosine kinase	Shaggy-related protein kinase alpha
BraA09g009840.3C	Membrane transport protein	Protein PIN-LIKES 7
BraA09g006170.3C	Protein kinase domain;; Protein tyrosine kinase;; Lipopolysaccharide kinase (Kdo/WaaP) family;; Phosphotransferase enzyme family	CDPK-related kinase 4
BraA09g007980.3C	RING-variant domain	--
BraA09g006730.3C	--	Protein BREAKING OF ASYMMETRY IN THE STOMATAL LINEAGE
BraA09g005740.3C	Plant self-incompatibility protein S1	S-protein homolog 13
BraA09g007310.3C	HEAT repeats;; HEAT repeat;; HEAT-like repeat	TORTIFOLIA1-like protein 3
BraA09g003840.3C	TPL-binding domain in jasmonate signalling;; Ethylene-responsive binding factor-associated repression;; Putative nuclear localisation signal	Ninja-family protein AFP3
BraA09g005120.3C	PWWP domain	--
BraA09g008490.3C	Ras family;; Ras of Complex, Roc, domain of DAPkinase;; ADP-ribosylation factor family	Ras-related protein RABH1a
BraA09g005980.3C	Sas10/Utp3/C1D family	--
BraA09g006310.3C	Voltage-dependent anion channel	S-type anion channel SLAH3
BraA09g008160.3C	--	--
BraA09g005280.3C	Protein phosphatase 2C	Probable protein phphatase 2C 73
BraA09g009320.3C	Lateral organ boundaries (LOB) domain	LOB domain-containing protein 36
BraA09g009130.3C	AP2 domain	Ethylene-responsive transcription factor ERF010

BraA09g006040.3C	FAD binding domain of DNA photolyase;; DNA photolyase	Cryptochrome DASH, chloroplastic/mitochondrial
BraA09g005320.3C	Kelch motif;; Kelch motif;; Galactose oxidase, central domain;; Kelch motif;; F-box domain;; Galactose oxidase, central domain;; F-box-like	F-box/kelch-repeat protein At5g26960
BraA09g008830.3C	AhpC/TSA antioxidant enzyme;; AhpC/TSA family	--
BraA09g007340.3C	Biotin/lipoate A/B protein ligase family;; Biotin protein ligase C terminal domain	Biotin--protein ligase 2
BraA09g009910.3C	AMP-binding enzyme	4-coumarate--CoA ligase-like 2
BraA09g008240.3C	Carbon-nitrogen hydrolase	Beta-ureidopropionase
BraA09g006400.3C	--	--
BraA09g009160.3C	Rapid ALkalinization Factor (RALF)	Protein RALF-like 34
BraA09g009760.3C	La domain	La-related protein 1B
BraA09g009930.3C	--	--
BraA09g008780.3C	--	Protein CROWDED NUCLEI 4
BraA09g005910.3C	--	--
BraA09g005220.3C	Pectinesterase;; Plant invertase/pectin methylesterase inhibitor	Putative pectinesterase/pectinesterase inhibitor 28
BraA09g007410.3C	1-deoxy-D-xylulose 5-phosphate reductoisomerase;; 1-deoxy-D-xylulose 5-phosphate reductoisomerase C-terminal;; DXP reductoisomerase C-terminal domain	1-deoxy-D-xylule 5-phphate reductoisomerase, chloroplastic
BraA09g010050.3C	1,3-beta-glucan synthase component;; 1,3-beta-glucan synthase subunit FKS1, domain-1;; Vta1 like	Calle synthase 5
BraA09g008950.3C	NPH3 family	BTB/POZ domain-containing protein NPY3
BraA09g005710.3C	--	--
BraA09g010100.3C	Adenylylsulphate kinase	Adenylyl-sulfate kinase 1, chloroplastic
BraA09g007040.3C	U-box domain	U-box domain-containing protein 52
BraA09g003940.3C	HAUS augmin-like complex subunit 5	AUGMIN subunit 5
BraA09g009680.3C	Auxin responsive protein	Auxin-responsive protein SAUR50
BraA09g008530.3C	VQ motif	--
BraA09g009540.3C	Mpp10 protein	--
BraA09g006990.3C	Terpene synthase family, metal binding domain;; Terpene synthase, N-terminal domain	Terpenoid synthase 25
BraA09g005870.3C	Cyclin, N-terminal domain;; Cyclin, C-terminal domain	Cyclin-A2-1
BraA09g009500.3C	Protein of unknown function, DUF547;; Leucine-zipper of ternary complex	--

	factor MIP1	
BraA09g008110.3C	--	--
BraA09g007600.3C	CRAL/TRIO domain;; Divergent CRAL/TRIO domain	Phphatidylinitol/phphatidylcholine transfer protein SFH6
BraA09g006270.3C	Glycosyl hydrolases family 18	Acidic endochitinase
BraA09g009410.3C	Zinc finger, C2H2 type	Protein indeterminate-domain 1
BraA09g005330.3C	SAM dependent carboxyl methyltransferase	Salicylate/benzoate carboxyl methyltransferase
BraA09g007870.3C	Bacterial transferase hexapeptide (six repeats)	Gamma carbonic anhydrase-like 1, mitochondrial
BraA09g009470.3C	Protein of unknown function (DUF677);; Protein BYPASS1-related	UPF0496 protein At5g66675
BraA09g005370.3C	--	--
BraA09g008910.3C	Adenylylsulphate kinase	Adenylyl-sulfate kinase 4, chloroplastic
BraA09g006280.3C	Peroxidase	Probable peroxidase 61
BraA09g007770.3C	Radical SAM superfamily	--
BraA09g006960.3C	PPR repeat family;; PPR repeat;; PPR repeat;; Pentatricopeptide repeat domain;; Pentacotripeptide-repeat region of PRORP;; Mitochondrial 28S ribosomal protein S27	Pentatricopeptide repeat-containing protein At5g61400
BraA09g006650.3C	Prefoldin subunit	Probable prefoldin subunit 5
BraA09g007220.3C	Embryo-specific protein 3, (ATS3);; PLAT/LH2 domain	Embryo-specific protein ATS3B
BraA09g009900.3C	AMP-binding enzyme	4-coumarate--CoA ligase-like 2
BraA09g006070.3C	Protein RETICULATA-related	Protein RETICULATA-RELATED 6, chloroplastic
BraA09g009180.3C	hAT family C-terminal dimerisation region;; Domain of unknown function (DUF4413)	Zinc finger BED domain-containing protein RICESLEEPER 2
BraA09g005110.3C	Cyclin C-terminal domain;; Cyclin, N-terminal domain;; Transcription factor TFIIB repeat	Cyclin-H1-1
BraA09g009010.3C	Subtilase family;; Peptidase inhibitor I9;; PA domain	Subtilisin-like protease SBT1.7
BraA09g005950.3C	--	Ethylene-responsive transcription factor ERF003
BraA09g009750.3C	3-dehydroquinate synthase;; Iron-containing alcohol dehydrogenase;; Iron-containing alcohol dehydrogenase	3-dehydroquinate synthase, chloroplastic
BraA09g005770.3C	GATA zinc finger	GATA transcription factor 12
BraA09g006410.3C	Transmembrane amino acid transporter protein;; Tryptophan/tyrosine permease family	Probable amino acid permease 7
BraA09g006010.3C	CCT motif;; B-box zinc finger	Zinc finger protein CONSTANS-LIKE 4
BraA09g006830.3C	--	Histone deacetylase 5

BraA09g008560.3C	--	--
BraA09g007570.3C	Glutaredoxin	Glutaredoxin-C1
BraA09g008060.3C	Inositol monophosphatase family	SAL1 phphatase
BraA09g005080.3C	EF hand associated;; Ras family;; EF hand associated;; Ras of Complex, Roc, domain of DAPkinase;; 50S ribosome-binding GTPase;; Dynamin family	Mitochondrial Rho GTPase 1
BraA09g005480.3C	Cupin;; Cupin domain	Probable germin-like protein subfamily 2 member 5
BraA09g007840.3C	Det1 complexing ubiquitin ligase;; SAP domain	--
BraA09g009480.3C	Mitochondrial calcium uniporter	Calcium uniporter protein 3, mitochondrial
BraA09g008250.3C	CHCH domain	--
BraA09g006820.3C	RNA recognition motif. (a.k.a. RRM, RBD, or RNP domain)	Glycine-rich RNA-binding protein 3, mitochondrial
BraA09g003930.3C	SRF-type transcription factor (DNA-binding and dimerisation domain);; K-box region	Truncated transcription factor CAULIFLOWER A
BraA09g005990.3C	Surp module;; RNA recognition motif. (a.k.a. RRM, RBD, or RNP domain);; cwf21 domain	Protein RRC1
BraA09g005010.3C	Ring finger domain;; RING-like zinc finger;; Zinc finger, C3HC4 type (RING finger);; Zinc finger, C3HC4 type (RING finger);; Anaphase-promoting complex subunit 11 RING-H2 finger	E3 ubiquitin-protein ligase ATL31
BraA09g009300.3C	NB-ARC domain;; Arabidopsis broad-spectrum mildew resistance protein RPW8;; Leucine Rich repeats (2 copies);; Leucine rich repeat;; ATPase family associated with various cellular activities (AAA)	Probable disease resistance protein At5g66900
BraA09g008200.3C	Sodium:sulfate symporter transmembrane region;; Citrate transporter	Dicarboxylate transporter 2.1, chloroplastic
BraA09g005860.3C	Permease family	Putative nucleobase-ascorbate transporter 10
BraA09g005060.3C	tRNA synthetase class II core domain (G, H, P, S and T);; Seryl-tRNA synthetase N-terminal domain	Serine--tRNA ligase, cytoplasmic
BraA09g006580.3C	HMG (high mobility group) box;; HMG-box domain	High mobility group B protein 7
BraA09g006670.3C	K-box region	Protein TRANSPARENT TESTA 16
BraA09g007930.3C	Protein kinase domain;; Protein tyrosine kinase	Cyclin-dependent kinase E-1
BraA09g008390.3C	EamA-like transporter family	WAT1-related protein At5g64700
BraA09g008750.3C	Reverse transcriptase (RNA-dependent DNA polymerase)	Putative ribonuclease H protein At1g65750
BraA09g009730.3C	Glycosyl hydrolases family 38 C-terminal domain;; Glycosyl hydrolases family 38 N-terminal domain;; Proteasome subunit;; Alpha mannosidase middle domain	Probable alpha-mannidase At5g66150
BraA09g006460.3C	WD domain, G-beta repeat	WD repeat-containing protein RUP2

BraA09g005750.3C	AUX/IAA family	Auxin-responsive protein IAA28
BraA09g006700.3C	Protein of unknown function (DUF1604);; Surp module	G patch domain-containing protein TGH
BraA09g010090.3C	PA domain;; Complement Clr-like EGF-like	Vacuolar-sorting receptor 3
BraA09g009020.3C	Subtilase family;; Peptidase inhibitor I9;; PA domain	Subtilisin-like protease SBT1.7
BraA09g005270.3C	Leucine Rich repeat;; Leucine Rich repeats (2 copies)	F-box/LRR-repeat protein 3
BraA09g008220.3C	NPH3 family	Root phototropism protein 3
BraA09g007300.3C	Plant self-incompatibility protein S1	S-protein homolog 25
BraA09g009620.3C	--	--
BraA09g005690.3C	WRKY DNA -binding domain	Probable WRKY transcription factor 50
BraA09g008350.3C	Pectinesterase;; Plant invertase/pectin methylesterase inhibitor	Probable pectinesterase/pectinesterase inhibitor 64
BraA09g006520.3C	JAB1/Mov34/MPN/PAD-1 ubiquitin protease;; Maintenance of mitochondrial structure and function;; Prokaryotic homologs of the JAB domain	26S proteasome non-ATPase regulatory subunit 14 homolog
BraA09g005620.3C	--	Probable membrane-associated kinase regulator 1
BraA09g009120.3C	Protein tyrosine kinase;; Protein kinase domain	Putative disease resistance RPP13-like protein 3
BraA09g009050.3C	Armadillo/beta-catenin-like repeat;; U-box domain;; Armadillo-like	U-box domain-containing protein 2
BraA09g005960.3C	--	Zinc finger protein 3
BraA09g009600.3C	--	Lipid phphate phphatase epsilon 2, chloroplastic
BraA09g008580.3C	Ras family;; Ras of Complex, Roc, domain of DAPkinase;; ADP-ribosylation factor family;; 50S ribosome-binding GTPase	Ras-related protein RABA4a
BraA09g008330.3C	MOZ/SAS family;; RNA binding activity-knot of a chromodomain	Histone acetyltransferase of the MYST family 1
BraA09g005700.3C	Possible lysine decarboxylase	Cytokinin ribide 5≈-monophphate phphoribohydrolase LOG8
BraA09g009460.3C	Oligosaccharyltransferase 48 kDa subunit beta	Dolichyl-diphphooligaccharide--protein glycyltransferase 48 kDa subunit
BraA09g007060.3C	--	--
BraA09g009960.3C	PLATZ transcription factor	--
BraA09g006080.3C	--	DNA-binding protein BIN4
BraA09g007100.3C	Brix domain	Peter Pan-like protein
BraA09g008150.3C	Ribosomal protein S28e	40S ribomal protein S28-2
BraA09g007800.3C	Adenylate kinase;; AAA domain;; Adenylate kinase, active site lid	Adenylate kinase 4
BraA09g006130.3C	--	--

Table S4 Primers used in this study

Primer	Primer sequence (5'-3')	Purpose
GOLDEN-F	ATGTCTTGTTTGGGTAGGATCTTG	Amplification of *BrGOLDEN* in cDNA and gDNA
GOLDEN-R	TCAAAGGAAAGGATCAATCCGAGG	
q-CRTISO-F	ATGGAAAGGCTGTGGGTGTAAG	QRT-PCR primers for the *BrCRITISO* (*BraA09g063710.3C*) gene in Chinese cabbage
q-CRTISO-R	GGCAATCTGTATCTGGAGGGAG	
q-ZDS-F	AACTCAAGCCCTATGACAAGC	QRT-PCR primers for the *BrZDS* (*BraA05g040310.3C*) gene in Chinese cabbage
q-ZDS-R	AAGGAACCAATCCGAGAAACT	
q-LCYB-F	AGGTGCTGGTGGTGCTTACTC	QRT-PCR primers for the *BrLCYB* (*BraA03g017230.3C*) gene in Chinese cabbage
q-LCYB-R	TTCCTGTGGTCCTTGTGGTTG	
q-VDE-F	ATTGTTTCACTTCACCTTGTC	QRT-PCR primers for the *BrVDE* (*BraA06g005700.3C*) gene in Chinese cabbage
q-VDE-R	GGATTTCGCAGTTACTCTTAG	
q-DXS-F	GAAGCAGAGGTAGACAAGGACGT	QRT-PCR primers for the *BrDXS* (*BraA01g021140.3C*) gene in Chinese cabbage
q-DXS-R	AAGAATAGATTGCACAGAAAGGC	
q-ZEP-F	TGGGTGTTGGGCTTGGTCC	QRT-PCR primers for the *BrZEP* (*BraA07g016880.3C* and *BraA07g016890.3C*) gene in Chinese cabbage
q-ZEP-R	GGCATAGCAATGTCAATGAAGAATC	
q-PSY-F	GTTTGAGTTTGTTGGGTGA	QRT-PCR primers for the *BrPSY* (*BraA02g006890.3C*) gene in Chinese cabbage
q-PSY-R	TCTTCTAACCTTGCTTCCC	
qGGPPS11.1-F	GCGAGGTGTGTTGGTTTGTTGTTTCA	QRT-PCR primers for the *BrGGPPS11.1* (*BraA08g021280.3C*) gene in Chinese cabbage
qGGPPS11.1-R	TTTCCCAGCAGTTTTCCCCAGTTCTT	
qGGPPS11.2-F	CGAAAGCTATAGGAACCGAA	QRT-PCR primers for the *BrGGPPS11.2* (*BraA01g002000.3C*) gene in Chinese cabbage
qGGPPS11.2-R	GTCATCAACCACCTGAAACA	
qBCH2-F	GAGGCTTGAGAAAGAGGAGAGGAAG	QRT-PCR primers for the *BrBCH2* (*BraA10g010930.3C*)

qBCH2-R	CCAATAAAATCGGTAGTAAACGGAC	gene in Chinese cabbage
qCYP97C1-F	AAGGACCGATCCCTAACGAAACAAA	QRT-PCR primers for the *CYP97C1* (*BraA07g021850.3.5C*) gene in Chinese cabbage
qCYP97C1-R	GAGAAAGACCGCAAGTGCCACTATT	
qCYP97A35-F	GCTTTTCCTTACTTACGGTGG	QRT-PCR primers for the *CYP97A3* (*BraA08g009810.3C*) gene in Chinese cabbage
qCYP97A3-R	AGCAGGTATGAGTCCTTTCCC	
q-ACTIN-F	GGAGCTGAGAGATTCCGTTG	QRT-PCR primers for the *BrACTIN* gene in Chinese cabbage
q-ACTIN-R	GAACCACCACTGAGGACGAT	
q-golden-F	GAGTCAAGTAGACCCAATAGTAGCGTCC	QRT-PCR primers for the *Brgolden* gene in non-golden head Chinese cabbage
q-golden-R	CACGTTGTTGACTTCAACAACCATCAAC	
q-GOLDEN-F	GAGTCAAGTAGACCCAATAGTGGCGTCA	QRT-PCR primers for the *BrGOLDEN* gene in golden head Chinese cabbage
q-GOLDEN-R	CACGTTGTTAACTTCAACTACCATCAAA	
HA-GOLDEN-F	gacgatgacgataagggatccATGTCTTGTTTGGGTAGGATCTTGT	Homologous arm primers used to construct *35S::BrGOLDEN* vector, Underscores indicate *BamH I* site and *Xba I* site, respectively.
HA-GOLDEN-R	tgcctgcaggtcgactctagaTCAAAGGAAGGGATCAATACGAG	
HA-GOLDENPro-F	gccaagcttgcatgcctgcaggGGGGTTCACATAGTGTTTCTTACAA	Homologous arm primers used to construct Pro$_{Brgolden}$:GUS vector, Underscores indicate *Sbf I* site and *BamH I* site, respectively.
HA-GOLDENPro-R	ggactgaccacccggggatccGGTTTCGAAGTCTTCAGCAGCA	
GOLDEN$_{Del}$-PBT3-N-F	ATCGAATTCCTGCAGGGCCATTACGGCCATGTCTTGTTTGG	Primers for Synthesis of Bait Plasmid GOLDENDel-PBT3-N in Membrane System Y2H
GOLDEN$_{Del}$-PBT3-N-R	AGCTACTTACCATGGGCCGAGGCGGCCTCAAAGGAAGGGATC	
GOLDEN$_{Ldel}$-PGBKT7-F	TCAGAGGAGGACCTGCATATGTCTTGTTTGGGTAGGATCTTG	Primers for Synthesis of Bait Plasmid GOLDENDel-PBT3-N in Nuclear System Y2H
GOLDEN$_{Ldel}$-PGBKT7-R	TCGACGGATCCCCGGGAATTCTCAAAGGAAGG	

	GATCAATAC	
golden-PBT3-N-F	ATCGAATTCCTGCAGGGCCATTACGGCCATGTCTTGTTTGG	Primers for Synthesis of Bait Plasmid golden-PBT3-N in Membrane System Y2H
golden-PBT3-N-R	AGCTACTTACCATGGGCCGAGGCGGCCTCAAAGGAAGGGATC	
PSY-PPR3-N-F	ACGATGTTCCAGATTACGCTGGATCCATGTCTTCTGTAGCAGTGTTATGGGTTGCTTCC	Primers for Synthesis of Bait Plasmid PSY-PPR3-N in Membrane System Y2H
PSY-PPR3-N-R	GTATCGATAAGCTTGATATCGAATTCTTAAGTTGTTCCTCTTGAACTTGGAGTCTTTAG	
PSY-PGADT7-F	GTACCAGATTACGCTCATATGTCTTCTGTAGCAGTGTTATG	Primers for Synthesis of Bait Plasmid PSY-PGADT7 in Nuclear System Y2H
PSY-PGADT7-R	CAGCTCGAGCTCGATGGATCCTTAAGTTGTTCCTCTTGAAC	
GLODEN$_{Ins}$-PBT3-N-F	TCGAATTCCTGCAGGGCCATTACGGCCATGTCTTGTTTGG	Primers for Synthesis of Bait Plasmid GLODEN$_{Ins}$-PBT3-N in Membrane System Y2H
GLODEN$_{Ins}$-PBT3-N-R	GCTACTTACCATGGGCCGAGGCGGCCTCAAAGGAAGGGATC	
goldenPro-F	GGGGTTCACATAGTGTTTCTTAC	Primers used to amplify *Brgolden* promoter fragments
goldenPro-R	CAAGATCCTACCCAAACAAGACAT	
